# Scrutinizing Mechanisms of the ‘Obesity Paradox in Sepsis’: Obesity Is Accompanied by Diminished Formation of Neutrophil Extracellular Traps (NETs) Due to Restricted Neutrophil–Platelet Interactions

**DOI:** 10.3390/cells10020384

**Published:** 2021-02-12

**Authors:** Iwona Cichon, Weronika Ortmann, Michal Santocki, Malgorzata Opydo-Chanek, Elzbieta Kolaczkowska

**Affiliations:** Department of Experimental Hematology, Institute of Zoology and Biomedical Research, Jagiellonian University, 30-387 Krakow, Poland; iwona.cichon@doctoral.uj.edu.pl (I.C.); weronika.ortmann@doctoral.uj.edu.pl (W.O.); michal.santocki@doctoral.uj.edu.pl (M.S.); malgorzata.opydo-chanek@uj.edu.pl (M.O.-C.)

**Keywords:** neutrophil extracellular traps, neutrophils, platelets, systemic inflammation, sepsis, endotoxemia, obesity

## Abstract

Systemic inflammation is a detrimental condition associated with high mortality. However, obese individuals seem to have higher chances of surviving sepsis. To elucidate what immunological differences exist between obese and lean individuals we studied the course of endotoxemia in mice fed high-fat diet (HFD) and ob/ob animals. Intravital microscopy revealed that neutrophil extracellular trap (NET) formation in liver vasculature is negligible in obese mice in sharp contrast to their lean counterparts (ND). Unlike in lean individuals, neutrophil influx is not driven by leptin or interleukin 33 (IL-33), nor occurs via a chemokine receptor CXCR2. In obese mice less platelets interact with neutrophils forming less aggregates. Platelets transfer from ND to HFD mice partially restores NET formation, and even further so upon P-selectin blockage on them. The study reveals that in obesity the overexaggerated inflammation and NET formation are limited during sepsis due to dysfunctional platelets suggesting their targeting as a therapeutic tool in systemic inflammation.

## 1. Introduction

Sepsis is “life-threatening organ dysfunction caused by a dysregulated host response to infection” [1]. In humans, the systemic inflammation carries a high risk of death up to 10–30% for sepsis and more than 40% for its most severe form, septic shock [2]. Reliable biomarkers of sepsis are lacking and there is no specific therapy. Therefore, sepsis remains a serious threat and urgent unmet medical need [3].

As sepsis develops secondary to microbial infection, it is characterized by the activation of leukocytes and endothelial cells, simultaneous activation of coagulation, and finally the cytokine storm [4]. Consequently, sepsis-associated deaths are rather not due to the microorganism itself, but to the persistent activation of the immune system [5]. It is now generally accepted that the balance between the systemic inflammatory response syndrome (SIRS) and the compensatory anti-inflammatory response (CARS) decides on sepsis outcome [1,5]. However, along with the inflammatory responses, alterations are also triggered in nonimmunologic pathways such as cardiovascular, metabolic, hormonal, neural as well as coagulation [1]. It is difficult to recapitulate such a diversity in animal models or computer simulations, nevertheless the former ones allow for an important input into our understanding of how immunity operates during systemic inflammation. Furthermore, intravital microscopy imaging (IVM) allows to monitor processes occurring directly in the vasculature of laboratory rodents. Thus far, with IVM, we and others showed that one of the highlights of the onset of sepsis is the formation of neutrophil extracellular traps (NETs) released by neutrophils [6,7,8,9]. This well reflects the clinical state as also in humans neutrophils are among major cellular participants of sepsis [10]. NETs are formed by highly activated granulocytes in which these structures are formed, and subsequently released extracellularly. They consist of decondensated DNA to which granular (e.g., neutrophil elastase, NE; high-mobility group protein 1, HMGB1) and nuclear (e.g., histones) proteins are attached, and their main function is to catch and immobilize pathogens [11]. IVM revealed that especially strong NET formation occurs in liver sinusoids, in comparison to lung [12], cecum [13], or skin [14] vasculature. Moreover, the excessive release of NETs causes liver injury, and in particular neutrophil elastase [15,16] and histones [7,17] of NET origin were shown to directly cause bystander cytotoxicity or contribute to microthrombosis. Moreover, their inappropriate formation and/or clearance further add to the pathology of sepsis [18]. Additionally, liver damage is independent of the inducing agent, be it Gram^−^ [9,19] or Gram^+^ [6,7] bacteria. Importantly, this reproduces well the course of sepsis in humans as liver failure complicating sepsis/septic shock has a significant impact on mortality [20].

Altered coagulation is one of the highlights of sepsis which is characterized by a drop in platelet count in circulation, reflecting platelet sequestration and their consumption in microthrombi [21]. With IVM it was shown that during lipopolysaccharide (LPS)-driven systemic inflammation in mice, platelets interact primarily with neutrophils already adherent within the liver sinusoids, and they form large aggregates on them [22]. We further proved the existence of a dynamic NET-platelet-thrombin axis in sinusoids regardless of the initiating bacterial stimulus (Gram^− OR +^ or bacterial product, LPS) [7]. 

A strong arm of biomedical research is based on studies of atypical cases or mutations as they often allow to identify the underlying mechanisms of a given disease (e.g., CCR5 delta 32 mutation vs. HIV entry [23]). In this light we hypothesized that the ‘obesity paradox in sepsis’ could provide such a platform for further understanding of systemic inflammation. Although both sepsis and obesity have high incidence and mortality rates, multiple epidemiological (e.g., [24,25,26]) analyses and empirical (e.g., [27,28]) studies on animal models suggest that obese individuals might be protected from sepsis-related mortality. Moreover, not only do obese patients seem to survive sepsis at a higher rate but also their long-term survival (> 1 year) is improved [29]. This is despite the fact that obese individuals are at greater risk of comorbidities such as diabetes, cardiovascular diseases, or liver abnormalities associated with shortened life expectancy [30]. However, not all studies confirm the very existence of such a paradox nor empirical studies minimizing bias and confounding factors were performed thus far [31,32].

The current study was not aimed to further scrutinize sepsis-related mortality but instead to compare the course of systemic inflammation in obese and lean mice focusing on neutrophils and their NETs as well as other cellular partners operating in the liver, the organ affected in both, sepsis [6,7] and obesity [33]. This is because hardly anything is known about differences in the immune response between the two in the course of sepsis as well as on possible causes of the paradox. The elevated adipose tissue mass and associated energy stores were proposed to be the causative factor due to the catabolic nature of sepsis [34]. On the other hand, as obesity is accompanied by a low-grade inflammation, altered course of systemic inflammation was suggested in obese individuals. Some data seem to confirm it as, for example, in obesity adipose tissue is the source of numerous inflammatory cytokines but surprisingly during sepsis obese patients have lower than expected level of proinflammatory cytokines [35,36].

Herein we report that neutrophils of obese mice, independent of the cause of obesity (overnutrition or genetics), have diminished capacity to cast NETs in vivo redundant of their weaker infiltration into the liver as the NET-to-neutrophil ratio confirms the weak trap release. In contrast, NET formation is unchanged by neutrophils isolated from such animals when NETs are induced ex vivo, implying that the faulty NET release in liver sinusoids is not intrinsic in nature. With IVM we further observed altered interactions of neutrophils with platelets, but not Kupffer cells, and their weaker aggregation. When we transferred platelets from mice kept on the control diet (ND) to those on a high fat diet (HFD), NET formation was partially restored. Therefore, the dysregulation was not related to platelet numbers but rather to their quality. Further blockage of P-selectin overexpressed on platelets from obese individuals resulted in further increase of NET formation ability by the ND recipients of HFD platelets. Overall, our data suggest that indeed, obesity attenuates sepsis severity by preventing overactivation of neutrophils and excessive NET formation due to impaired platelet–neutrophil interactions. 

## 2. Materials and Methods

### 2.1. Diet- and Genetic-Driven Models of Obesity

Three weeks old C57BL/6J male mice were purchased from Charles River Laboratories (Sulzfeld, Germany; via AnimaLab). Mice were randomly divided into two groups and were fed either a control diet (= normal diet, ND; Altromin, C1000, Fat 13%, Carbohydrates 67%, Protein 20% of kcal) or a high-fat diet (HFD; Altromin, C1090 – 60, Fat 60%, Carbohydrates 24%, Protein 16% of kcal) [6]. The food and tap water were available ad libitum. Mice were kept on either diet for at least 12 weeks and during this time their body mass significantly increased, and it was higher by 54.68 ± 14.53% than that of their lean counterparts by week 12 (Supplementary Figure S1A). Mice were weighed once a week, their food and water intake was monitored throughout the study (Supplementary Figure S1B,C).

Male mice with leptin-deficiency (ob/ob) on the C57BL/6J gene background (B6.Cg-Lep<ob>/J) were purchased from Charles River Laboratories (Calco, Italy; via AnimaLab) at 6 weeks of age and fed the recommended chow diet ad libitum (SAFE, Scientific Animal Food and Engineering; D5K52 CRL). Food and tap water were available ad libitum. Mice were weighed once a week and food and water intake were monitored continuously. Mice were used in experiments at the age of 12 weeks. Age matched C57BL/6J mice (wild type, wt) served as controls. All experimental animal protocols were approved by the Local Ethical Committee No. II in Kraków (293/2017) and were in compliance with the EU Animal Care Guidelines. 

### 2.2. Systemic Inflammation Models: Endotoxemia and *S. aureus*-Induced Sepsis

Mice were intraperitoneally (i.p.) injected with 1 mg/kg per body weight (b.w.) LPS (*Escherichia coli* serotype 0111:B4; Sigma-Aldrich, Saint Louis, MO, USA) in saline to induce endotoxemia [9], and they were subjected to intravital imaging at various time points post LPS injection as indicated in each figure. Some animals were left untreated. In selected experiments, *Staphylococcus aureus*-induced sepsis was studied. Methicillin-resistant *Staphylococcus aureus* (MRSA, US-300, clinical isolate) was stored in tryptic soy broth (TSB, Sigma Aldrich, Saint Louis, MO, USA) containing glycerol (50% v/v) at −80 °C. Cultures were inoculated from stocks into 10 mL media. Bacteria were grown overnight under constant rotation (180 rpm) to mid-logarithmic growth phase at 37 °C, centrifuged at 5000× *g* for 5 min, washed and then resuspended in saline to the desired OD (600 nm). Bacteria were then injected via the tail vein (1–2 × 10^7^ CFUs, 200 μL/ mouse). MRSA was a kind gift from Dr. Joanna Koziel (Department of Microbiology, Faculty of Biochemistry, Biophysics and Biotechnology, Jagiellonian University, Krakow, Poland). 

In some experiments, mice were at first subjected to intravital imaging and LPS was administrated while imaging via the jugular vein. Subsequently, the mice were imaged. Prior to experiments not involving IVM, mice were anesthetized with a mixture of ketamine hydrochloride (200 mg/kg b.w.; Biowet Pulawy, Pulawy, Poland) and xylazine hydrochloride (10 mg/kg b.w.; aniMedica, Südfeld, Germany) and their blood was collected by cardiac puncture in a heparinized syringe. Samples were centrifuged at 1200× *g* for 10 min for the retrieval of plasma and frozen at −20 °C prior to further analyses. In some mice, subsequently the peritoneal cavity was lavaged with 1.5 mL saline, and after a 30 s gentle manual massage, 1 mL exudate was retrieved and frozen at −20 °C prior to subsequent assessment. Once mice were sacrificed their organs were dissected and weighed.

### 2.3. ADAMTS13 and DNase Treatment

In some experiments, 4 h after induction of endotoxemia mice were intravenously (i.v.) injected (via tail vein) with a mixture of DNase 1 (1000 U per mouse; Roche, Basel, Switzerland) and ADAMTS13 (3 μg per mouse; R&D Systems, Minneapolis, MN, USA) [6]. After next 2 h blood was collected via the heart puncture as described above. 

### 2.4. Recombinant Leptin and Leptin Neutralizing Antibody Treatments

Lyophilized mouse recombinant leptin was purchased from Peprotech (Cranbury, NJ, USA) and it was reconstituted in sterile water. Some ND and HFD mice received an intraperitoneal injection of recombinant leptin in saline at 1 mg/kg b.w., at 5:00 AM or 8:00 PM [37]. Control groups were injected with 0.9% saline. After 12 h since the leptin injection, endotoxemia was induced by i.p. administration of LPS. Twenty-four hours following LPS challenge intravital microscopy was performed. Some groups of ND and HFD mice received i.v. leptin neutralizing antibodies (Mouse Leptin/OB Antibody, polyclonal goat IgG, R&D Systems, Minneapolis, MN, USA) via tail vein (3 µg/mouse in 0.9% saline) at 12:00 AM or 6:00 PM, and after 30 min since the induction of endotoxemia (1 mg/kg b.w. LPS, i.p.) [38]. Control groups were injected intravenously with polyclonal goat IgG isotype control antibody (R&D Systems, neutralizing antibodies (Mouse Leptin/OB Antibody, polyclonal goat IgG, R&D Systems, Minneapolis, MN, USA). The mice were imaged at 24 h of endotoxemia. In the above experiments, we took into account the circadian rhythmic expression of leptin as circulating plasma leptin levels are fluctuating over the 24 h day [37]. 

### 2.5. CXCR2 Chemokine Receptor Antagonist Treatment 

CXCR2 was blocked using its potent and selective antagonist SB225002 (Tocris, Bristol, UK). Mice were injected i.p. with the compound at 10 mg/kg b.w. 30 min before endotoxemia induction (1 mg/kg b.w. LPS, i.p.). The mice were imaged at 24 h of systemic inflammation.

### 2.6. IL-33 Neutralizing Antibody 

Neutralizing monoclonal antibody against mouse IL-33 (Bondy-1-1, AdipoGen, San Diego, CA, USA) was injected intraperitoneally (14 µg/mouse; in 0.9% saline) 30 min before endotoxemia induction (1 mg/kg b.w. LPS, i.p.). Control mice received the same dosage of isotype control antibody (Mouse IgG2b (BPC4), Ancell, Stillwater, MN, USA) before LPS challenge. Mice were imaged at 24 h of systemic inflammation.

### 2.7. Preparation of the Mouse Liver for Intravital Microscopy

Prior to IVM, mice were anesthetized with a mixture of ketamine hydrochloride (200 mg/kg b.w.) and xylazine hydrochloride (10 mg/kg b.w.). Subsequently, cannulation of the right jugular vein was performed to facilitate supply of the anesthetics/antibodies/dyes/LPS. Preparation of the liver for intravital imaging was performed as previously described [6]. 

### 2.8. Preparation of the Adipose Tissue for Intravital Microscopy

Mice were anesthetized by intraperitoneal injection with the xylazine-ketamine mixture and cannulation of right jugular vein was performed as described above. Visceral white adipose tissue was prepared as follows; a midline incision followed by a lateral incision along the costal margin to the midaxillary line was performed to expose the adipose tissue. The mouse was placed in a right lateral position, and the adipose tissue was externalized onto an imaging board using saline-soaked cotton swabs without touching the part of adipose tissue, which underwent imaging. Finally, two lines of Vaseline (Aflofarm, Pabianice, Poland) were applied, one for each of the two opposite sides of the coverslip and the adipose tissue was covered with a coverglass. The space underneath the coverglass was filled with saline (creating a wet chamber) to keep the organ moist throughout the time of imaging.

### 2.9. Spinning Disk Confocal Intravital Microscopy (SD-IVM) 

The livers were imaged with a ZEISS Axio Examiner.Z1 upright microscope equipped with a metal halide light source (AMH-200-F6S; Andor, Oxford Instruments, Abingdon, UK) with motorized 6 position excitation filter wheel and laser-free confocal spinning disk device (DSD2; Andor, Oxford Instruments, Abingdon, UK) with ZEISS EC Plan-NEOFLUAR 10×/0.3 and/or ZEISS EC Plan-NEOFLUAR 20×/0.5 air objective. The following filters were used, four excitation filters (DAPI: 390/40 nm; GFP: 482/18 nm; RFP: 561/14 nm; Cy5: 640/14 nm) and appropriate emission filters (DAPI: 452/45 nm; GFP: 525/45 nm; RFP: 609/54 nm; Cy5:676/29 nm). For fluorescence detection, the 5.5 megapixel sCMOS camera (Zyla 5.5; Andor, Oxford Instruments, Abingdon, UK) was used and the iQ 3.6.1 acquisition software (Andor, Oxford Instruments, Abingdon, UK) to drive the microscope. 

### 2.10. 3D Reconstruction of Liver Cross-Sections 

Overview of mouse livers was established on a side view of a 3D reconstruction (IMARIS v8.4.2 software, Bitplane, Oxford Instruments, Abingdon, UK) of a series of optical cross-sections (z stacks). In brief, a series of z stacks through the liver was performed with a z-step of 1 µm approximately through 50 z planes. Then, liver structure was reconstructed with IMARIS v8.4.2 software with DAPI and RFP channels, for neutrophils and platelets, respectively

### 2.11. NET Formation Analyses In Vivo

Imaging of NET components was performed with intravital immunofluorescence analysis. Namely, NETs were visualized by costaining of neutrophil elastase (1.6μg/mouse; Alexa Fluor 647 anti-neutrophil elastase antibody, clone G-2, Santa Cruz Biotechnology, Dallas, TX USA), histones H2A.X (0.5µg/mouse; AF555 anti-H2A.X antibody, clone 938CT5.1.1, Santa Cruz Biotechnology, Dallas, TX USA), and extracellular DNA (0.1 mM in 0.9% saline; Sytox green, Invitrogen, Carlsbad, CA, USA). All antibodies were injected i.v. via the jugular vein ≈20 min prior to intravital imaging. Sytox green was administrated during imaging as it stains DNA instantly. NETs were quantified with SD-IVM using previously published approach [6,39]. Quantitative data on NET components are expressed as the percentage of area in each field of view (FOV) covered by positive fluorescence staining. In some analyses NET-to-neutrophil ratio was calculated based on NE data and neutrophil numbers.

### 2.12. Neutrophil, Kupffer Cell and Platelet Quantification In Vivo

Neutrophils were visualized with anti-mouse Ly6G antibodies (1.6 μg/mouse; Brilliant Violet 421 anti-Ly6G, 1A8, BioLegend, San Diego, CA, USA) and Kupffer cells (KCs) were stained with eFluor 660 anti-F4/80 (1.6 μg/mouse, clone BM8; eBioscience, San Diego, CA, USA). Platelets were visualized with anti-CD49b antibodies (1.2 μg/mouse; PE anti-CD49b clone HMα2, BioLegend, San Diego, CA, USA). Neutrophils and KCs were counted per 20× FOV, minimum 5 FOV from each mouse. The area covered by platelets was estimated as described previously [6,39]. For platelet counts, thresholded images were converted to binary (black and white), and the area per field of view covered by positive fluorescence staining (black = platelets) was calculated with ImageJ v1.53a software. Neutrophils counts in peritoneal exudate were done with a hemocytometer following staining with Türk solution (0.01% crystal violet in 3% acetic acid; both Sigma-Aldrich, Saint Louis, MO, USA).

### 2.13. Estimation of Platelet Interactions with Neutrophils and Kupffer Cells

For analysis of platelet interactions with neutrophils and Kupffer cells, single frames of videos were analyzed. Interactions were considered to occur when platelets adhered to leukocytes for at least 6 s. Previously, transient “touch-and-go” interactions of platelets and KCs in basal conditions were estimated as briefer than 1 s as opposed to sustained adhesion [39]. Formation of platelet aggregates on leukocytes was studied by comparison of 10 subsequent frames (6 s apart covering 60 s) from each video. For evaluation of platelet aggregates, thresholded images were converted to binary, and the area per field of view covered by positive fluorescence staining was calculated with ImageJ v1.53a software. 

### 2.14. Platelets Isolation and Transfusion 

A syringe with a 27 G needle containing 3.8% sodium citrate (200 µL/mL blood, Chem-Lab NV, Zedelgem, Belgium) was used to puncture the heart and approx. 1 mL of blood was collected from each mouse. Blood was diluted in PBS (1:1 ratio) and centrifuged at 350× *g* for 5 min at RT. Then, 5–10 min after centrifugation, the upper layer (platelet rich plasma, PRP) was collected. Subsequently, 2 mM EDTA (final concentration) was added to PRP, and tubes were centrifuged at 200× *g* for 5 min at RT. The upper layer of PPP (platelet poor plasma) was discarded and platelets were washed in PBS and then pelleted by centrifugation at 1000× *g* for 15 min at RT. The pellet of platelets was resuspended in sterile 0.9% saline. Platelets were counted manually with a hemocytometer and adjusted to density of 6–7 × 10^7^/200 µL saline and injected i.v. to the respective groups of mice. The following groups were designed: platelets isolated from lean mice and injected to lean mice (ND → ND), platelets isolated from lean mice and injected to obese mice (ND → HFD), platelets isolated from obese mice and injected to lean mice (HFD → ND), and platelets isolated from obese mice and injected to obese mice (HFD → HFD). Immediately after platelets transfusion, mice were injected with LPS (1 mg/kg b.w. LPS, i.p.). Control groups received i.v. 0.9% saline and then endotoxemia was induced. Mice were imaged at 24 h of systemic inflammation. 

### 2.15. P-Selectin Blocking Experiments

In the blocking experiments, platelets from obese mice were isolated as described above and then incubated with blocking rat anti-mouse CD62P antibody (5 µg/mL, clone RB40.34 (RUO); BD Bioscience, San Jose, CA, USA) for 20 min at RT. After the incubation, platelets were washed and suspended in sterile saline. They were injected intravenously into lean mice that were immediately injected with LPS (1 mg/kg b.w. LPS, i.p.). The mice were imaged at 24 h of systemic inflammation.

### 2.16. NET Formation Analyses Ex Vivo

Bone marrow neutrophils were isolated from mice as described previously [19]. The purity and viability of isolated neutrophils was over 98–99% in each experiment as estimated by Türk solution and Trypan blue dye (Sigma-Aldrich, Saint Louis, MO, USA), respectively. 

Subsequently neutrophils were let to adhere for 30 min on coverglasses and then they were stimulated for 6 h with LPS (75 μg/mL, final concentration). Immediately after incubation, the cells were carefully fixed in sequence of 1%, 2%, and 3% paraformaldehyde in PBS for 2, 10, and 20 min, respectively, to not disrupt formed NETs and then they were washed in PBS. Subsequently the preparations were immunocytostained for the presence of NETs with rabbit polyclonal anti-histone H3 (citrulline R2 + R8 + R17) antibodies diluted 1:200 in 1% BSA/PBS (Abcam, Cambridge, UK) and incubated overnight at 4 °C in a humid chamber. The slides were then washed in PBS and incubated with Cy3-conjugated goat anti-rabbit IgG (H + L) antibody (diluted 1:300 in PBS/1% BSA, Jackson Immunoresearch, Ely, UK) for 1 h at RT. At the end of the procedure, Sytox green was added to stain for extDNA (5 μM). After washing in PBS the coverglasses were mounted with VECTASHIELD Mounting Medium (Vector Laboratories, Burlingame, CA, USA). NETs stained on coverglasses were visualized using the ZEISS Axio Examiner.Z1 equipped with DSD2 as in in vivo studies. ImageJ v1.53a was used to convert images to a grayscale (8-bit type) and thresholded to black and white to estimate the positive signal of extDNA/NET components (black) and expressed as percentage of FOV covered area.

### 2.17. Neutrophil Elastase Activity 

The activity of free neutrophil elastase in the samples of peritoneal fluid was assessed with a chromogenic substrate: N-methoxy-succinyl-Ala-Ala-Pro-Val-p-nitroanilide (Sigma-Aldrich, Saint Louis, MO, USA) dissolved in 1-methyl-2-pyrrolidinone (Sigma-Aldrich, Saint Louis, MO, USA). Samples of the peritoneal fluid (100 µL each) were placed on 96-well transparent plate and 100 µL of 0.2 M Tris-HCl buffer (pH 8.0) containing 0.2% BSA (Sigma-Aldrich, Saint Louis, MO, USA) was added to each sample. Simultaneously, a standard curve was prepared using elastase from human leukocytes (Sigma-Aldrich, Saint Louis, MO, USA). Next, 10 µL of substrate specific for neutrophil elastase was added to each well and the optical density was read with a Microplate Reader (Tecan, Infinity F200 Pro, Männedorf, Switzerland) at 405 nm in different time points (0, 1, and 24 h) after incubation at 37 °C. The NE activity was calculated from the standard curve and expressed in units/mL (one unit releases one nanomole of p-nitrophenol/s from BOC-L-alanine pnitrophenyl ester at pH 6.5 at 37 °C).

### 2.18. Alanine Transaminase (ALT) Activity

Blood from anaesthetized mice was collected by cardiac puncture with a heparinized syringe. Blood samples were centrifuged at 1200× *g* for 10 min at 4°C for the plasma retrieval. Plasma samples were analyzed for ALT levels as per manufacturer’s protocol (Sigma-Aldrich, Saint Louis, MO, USA). ALT activity was determined with a Microplate Reader (Tecan, Infinity F200 Pro, Männedorf, Switzerland) at 570 nm and expressed in nM/min/mL (one unit of ALT was defined as the amount of enzyme that generates 1.0 μM of pyruvate per minute at 37 °C).

### 2.19. Triglyceride, Cholesterol, Glucose, and Leptin Levels

Total plasma triglycerides and cholesterol levels were quantified with colorimetric Triglyceride Quantification assay and Cholesterol Quatification kit (both Sigma-Aldrich, Saint Louis, MO, USA), respectively. Measurement of plasma leptin levels was performed using enzyme-linked immunosorbent assay (Sigma-Aldrich, Saint Louis, MO, USA). All procedures were carried out as per manufacturer’s protocol. For glucose level estimation, blood was obtained from the tail vein by cutting off 2 mm of the tip with a sterile single use scalpel. Measurements were performed with the Bayer Contour (Ascensia Entrust, Mishawaka, IN, USA) glucose meter.

### 2.20. Flow Cytometry

Employing flow cytometry (FACSCalibur, Becton Dickinson, San Jose, CA, USA), neutrophils isolated from bone marrow were gated as Ly6G^+^ cells (PE anti-mouse Ly6G, 1A8, BioLegend, San Diego, CA, USA) and then LFA-1^+^ (PE anti-mouse CD11a/CD18, BioLegend, San Diego, CA, USA) neutrophils were quantified within this population using CellQuest Pro Pro v5.2.1 software (Becton Dickinson, San Jose, CA, USA). κ isotype control (PE Rat IgG1, RTK2071, BioLegend, San Diego, CA, USA) was run in parallel. In experiments verifying purity of isolated platelets, the following antibodies were used—anti-mouse PE CD41 antibody (clone MWReg30; BioLegend, San Diego, CA, USA), PE Ly-6G antibody (clone 1A8-Ly6g; eBioscience, San Diego, CA, USA), PE F4/80 antibody (clone BM8; eBioscience, San Diego, CA, USA), Alexa Fluor 647 Ly-6G antibody (clone 1A8; BioLegend, San Diego, CA, USA), PE IgG2a, κ isotype control antibody (clone MOPC-173; BioLegend, San Diego, CA, USA), PE IgG2a, κ isotype control antibody (clone RTK2758; BioLegend, San Diego, CA, USA).

### 2.21. Cytokine Measurement 

The blood plasma content of mouse IL-1β and IL-6 was measured by Mouse IL-1β ELISA Ready-SET-Go (Affymetrix, ThermoFisher Scientific, Waltham, MA, USA) and Mouse IL-6 ELISA Ready-SET-Go (eBioscience, ThermoFisher Scientific, Waltham, MA, USA). The assays were carried out as indicated by the manufacturers.

### 2.22. Statistical Analyses

All data are presented as mean values ± SD. Data were compared both by unpaired two-tailed Student’s *t*-test and one-way analysis of variance with Bonferroni multiple comparisons post hoc test. Statistical significance was set at *p* < 0.05.

## 3. Results

### 3.1. Metabolic Shift in Obese Mice

Some metabolic parameters differed between obese and lean animals and they included increased blood glucose and cholesterol levels (Supplementary Figure S2A,B). Triglyceride (TGA) levels did not differ between untreated ND and HFD mice by week 12 (Supplementary Figure S2C; opening time point, marked as 0 h of sepsis). However, in the course of sepsis TGA levels increased earlier in obese animals on HFD (peak at 3 h) and declined by 24 h of sepsis. Whereas in lean mice (ND) the TGA increased slower and peaked at 24 h (Supplementary Figure S2C). This is in line with other studies on mice fed HFD which develop many of the characteristics of the metabolic syndromes, including accumulation of the adipose tissue (especially visceral), higher glucose and cholesterol levels, fatty livers (please see below) and their triglyceride levels are fluctuating differently upon systemic inflammation [40,41]. Of note, endotoxemia differentially affected body weight, namely mice on HFD lost significantly less weight than ND-fed animals upon LPS inoculation (Supplementary Figure S2D; expressed as percentage of weight loss).

### 3.2. Liver Is Affected in Obese Mice

Spleens of the obese mice, both due to the HFD and a genetic mutation (ob/ob mice), were of the same weight as those of lean controls while their kidneys and especially livers were significantly heavier (Supplementary Figure S3A). Livers were not only significantly bigger (by app. 30%) but also damaged as visualized by their altered morphology (Supplementary Figure S3B) and elevated levels of alanine transaminase (ALT) (Supplementary Figure S3C). The morphology presented itself as steatosis with macrovesicular fatty degeneration (multiple fat droplets) of the liver cells, and hepatocyte ballooning. Occasionally large vacuoles or even fatty cysts were visible in hepatocytes (optically “empty” spaces, circular black structures seen in Supplementary Figure S3B). 

### 3.3. Formation of NETs in Liver Sinusoids Is Impaired in Obese Mice

**Figure 1:**
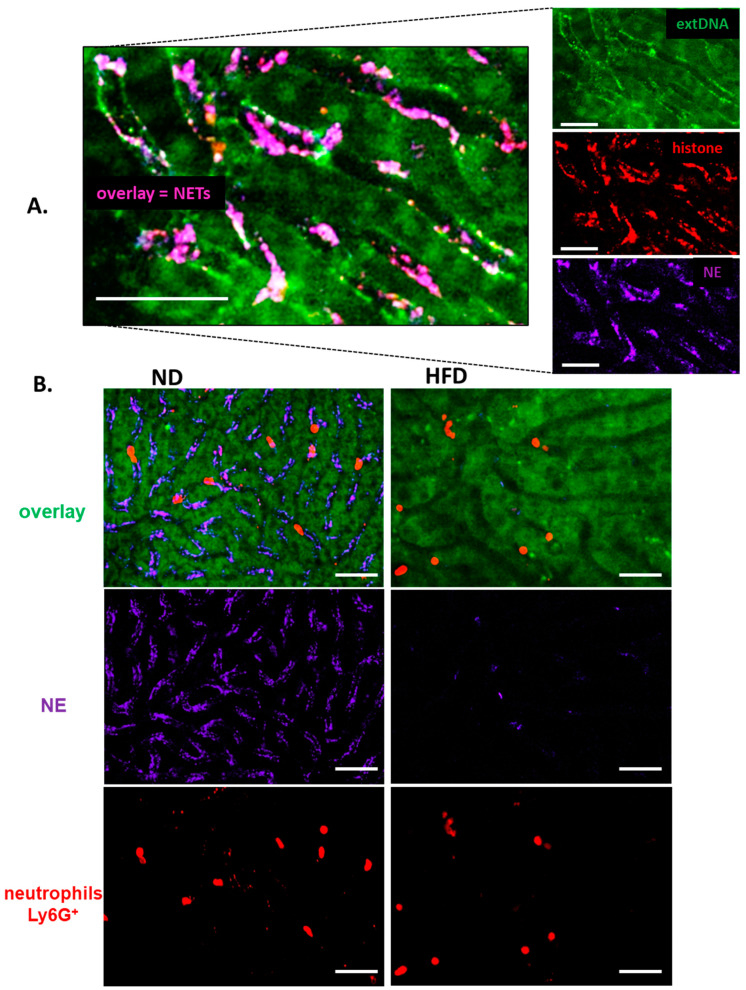
Deposition of neutrophil extracellular traps (NETs) in liver sinusoids during endotoxemia in lean (ND, left) and obese (HFD, right) mice. (**A**) Representative images of NETs were acquired with Spinning Disk Confocal Intravital Microscopy (SD-IVM) at 24 h of endotoxemia. To visualize colocalization of NET components, the frames from each channel were overlaid. On images autofluorescent hepatocytes (dim green) can be observed in between which sinusoids are localized (black ducts). In the latter structures, neutrophil elastase (NE, violet), histone H2A.X (red), and extracellular DNA (extDNA, bright green) overlaying signal is visible lining along endothelium. (**B**) Representative images revealing differences in NET deposition (NE and extDNA) and neutrophil numbers in sinusoids of ND and HFD mice at 24 h of endotoxemia. The scale bar indicates 50 μm.

Throughout the systemic inflammation, in either diet-induced or genetically driven obese mice, only negligible quantities of NETs were detected in liver sinusoids in comparison to their lean littermates (Figure 1B, Supplementary Figures S4A,B and S5A,C). NETs were identified as structures composed of extracellular DNA (extDNA) and neutrophil elastase (NE) or histones H2A.X (Figure 1A,B). Additionally, presence of histone H2A.X was confirmed in their structure (Figure 1A, Supplementary Figure S6).

**Figure 2:**
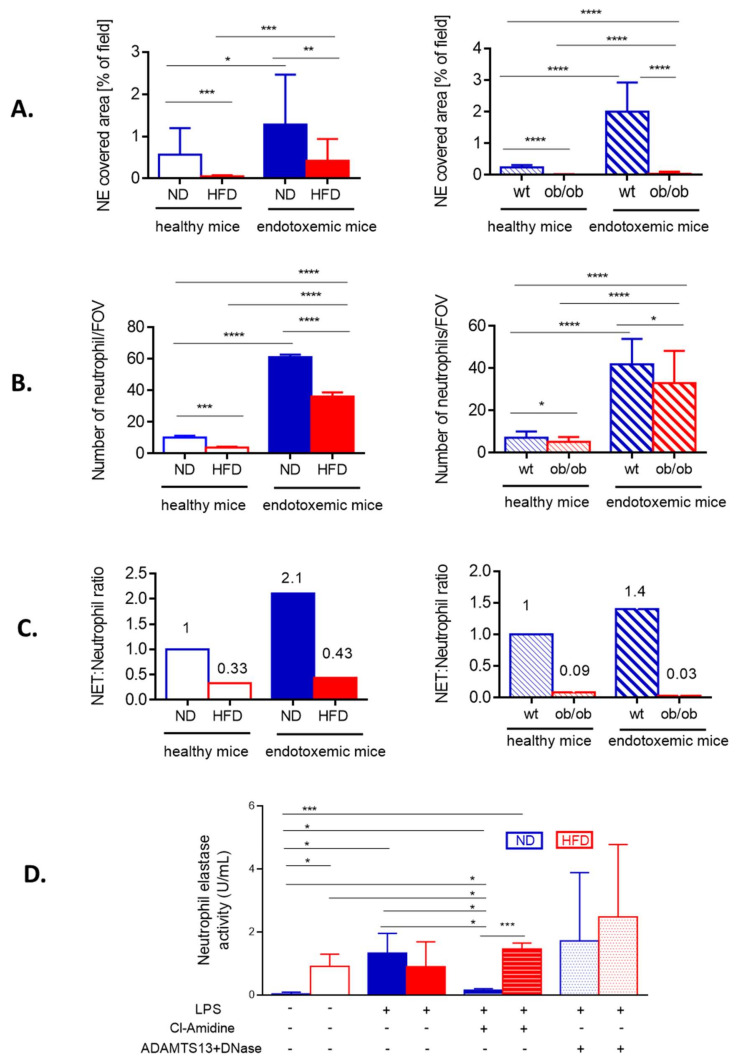
Quantification of neutrophil extracellular trap (NET) formation and neutrophil accumulation in liver sinusoids during endotoxemia in lean and obese mice. The analyses were performed in both models of obesity, in high fat diet (HFD) animals and their controls (ND) (left column), and ob/ob mice and their wild-type counterparts (wt) (right column). (**A**) Quantitative analysis of NETs within the liver sinusoids: area (%) covered by neutrophil elastase (NE). (**B**) The numbers of infiltrating neutrophils were quantified with ImageJ v1.53a software and are expressed as number per field of view (FOV). (**C**) Ratio of NETs to neutrophils was calculated by dividing % of formed NETs by number of neutrophils. A 1 represents arbitrary value for NET formation by neutrophils of healthy either ND (left panel in C) or wt mice (right panel in C). (**D**) Activity of neutrophil elastase (NE) was measured in blood plasma. The legend under the graph (LPS, Cl-Amidine, ADAMTS13+DNase) refers to treatments received by animals to either induce endotoxemia (lipopolysaccharide, LPS), to block NET formation (Cl-amidine) or to detach NETs from the vasculature (ADAMTS13+DNase). Asterisks indicate significant differences between lean (either ND or wt) and obese (either HFD or ob/ob) mice using unpaired two-tailed Student’s *t*-test (* *p* ≤ 0.05, ** *p* ≤ 0.01, *** *p* ≤ 0.001, **** *p* ≤ 0.0001). Data are shown as mean ± s.d.; *n* ≥ 3 per group.

The weak NET formation during LPS-induced systemic inflammation was independent of the obesity type, be it high fat diet-induced (HFD) or genetic (ob/ob mice), and is clearly visible on images presented in Figure 1 (HFD) and Supplementary Figure S4 (ob/ob animals). Data quantification revealed that even at the baseline levels (“healthy mice” in Figure 2A) numbers of NETs differed between obese (both diet-based and genetic-driven) and lean individuals but the difference was especially apparent upon sepsis induction and its progression (“endotoxemic mice” in Figure 2A, and Supplementary Videos S1 and S2). Of note, NET release was very low in either model of obesity at any studied time point (kinetics of NET formation: Supplementary Figure S5A,C). The same data on NETs was obtained if either NE or H2A.X levels were evaluated (Figure 2A vs. Supplementary Figure S6A–C). To verify, if the observed phenomenon is endotoxemia-specific or more general, sepsis was also induced by inoculation of MRSA. Data presented in Supplementary Figure S7A,B clearly shows that also in the course of Gram^+^-sepsis obese mice form less NETs, although overall the NET release is stronger upon *S. aureus* inoculation, than during endotoxemia.

In order to verify if weak NET formation could simply result from lower neutrophil infiltration, the cell counts in liver sinusoids were performed in endotoxemic mice. There were indeed significantly less neutrophils present in the vasculature of HFD and ob/ob mice (Figure 2B), but the ratio of NETs-to-neutrophils shows that the cells of obese individuals released less NETs independently of their numbers (Figure 2C). The lack of correlation between NET release and neutrophil numbers in liver sinusoids was observed at any studied time point in obese mice (Supplementary Figure S5B,D). The data from intravital imaging was verified by the measurement of NE activity in blood plasma. Elevated levels of soluble NE are well recognized in obesity [42] and in line with this, we detected active NE in plasma of otherwise healthy (no LPS) HFD mice (Figure 2D). During sepsis (24 h) it increased only in ND, but not HFD mice, and its origin from NETs was confirmed by almost complete depletion of the activity when Cl-amidine, the NET inhibitor, was used (Figure 2D). In HFD animals, levels of active NE were unchanged upon inhibition of NET formation. Next we attempted to dissect the activity of soluble NE from that of NET-NE and pretreated some mice with a cocktail of ADAMTS13 and DNase I. The latter enzyme dissolves DNA structure [11] whereas ADAMTS13 cleaves von Willebrand factor lining endothelium to which NET components secondarily attach [6]. 

With this approach we aimed to liberate NE from NET and/or endothelium. Although we observed a tendency to higher NE activity in both groups (ND/HFD) of mice, suggestive of successful NE detachment from NETs, because of a broad distribution of data, we did not observe statistically significant differences (Figure 2D). Nevertheless, our data confirms that there are less NETs formed by neutrophils of obese individuals and this phenomenon cannot be explained by weaker neutrophil infiltration.

### 3.4. Neutrophil Counts in the Adipose Tissue and Blood versus Peripheral Tissues

**Figure 3:**
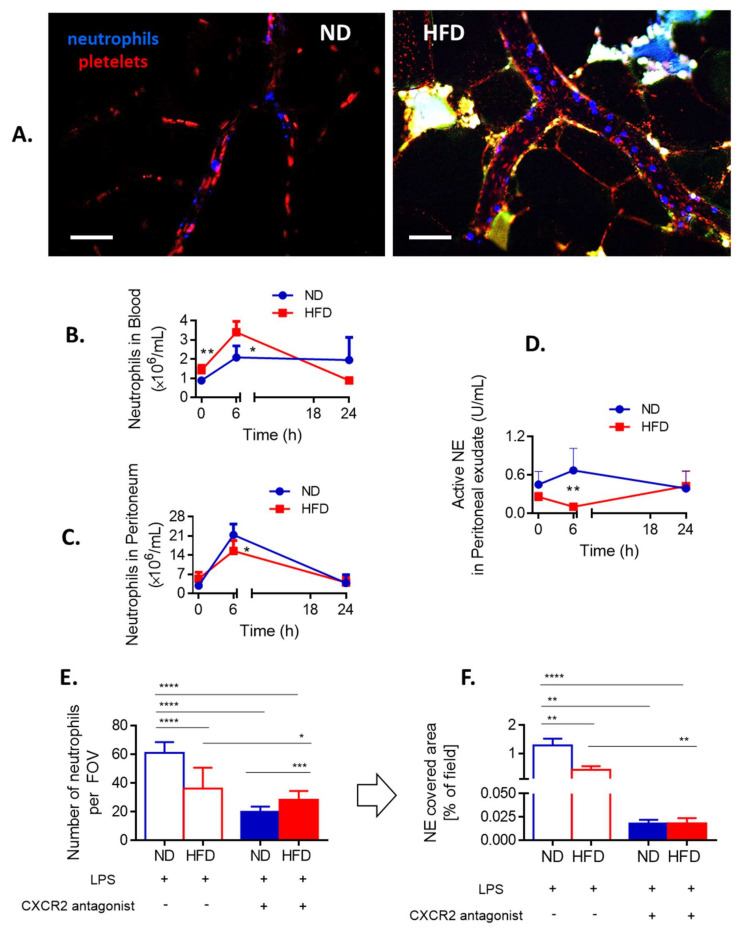
Neutrophil infiltration of the adipose tissue and peripheral tissues, and CXCR2-dependency of neutrophil accumulation in the sinusoids of lean (ND) and obese (HFD) mice. (**A**) Representative images of the vasculature in the adipose tissue of ND and HFD animals. To visualize blood flow, platelets were stained (red) and neutrophils labelled in blue. Autofluorescent adipose tissue is seen as white/green as the background signal. The scale bar indicates 50 μm. Neutrophil counts in blood (**B**) and peritoneal lavage (**C**) were performed with a hemocytometer at different time points of endotoxemia. Additionally, neutrophil elastase (NE) activity was evaluated in the peritoneal exudate (**D**). To verify if neutrophil infiltration of liver sinusoids depends on a chemokine receptor CXCR2 some ND and HFD mice were pretreated with its antagonist (SB225002) and 24 h after induction of endotoxemia neutrophil infiltration (**E**) as well as neutrophil extracellular trap (NET) formation (**F**) in the liver vasculature were estimated. In E-F blue line/filling is always used for NDs and red for HFD mice. Asterisks indicate significant differences between lean (either ND or wt) and obese (either HFD or ob/ob) mice using unpaired two-tailed Student’s *t*-test (* *p* ≤ 0.05, ** *p* ≤ 0.01, *** *p* ≤ 0.001, **** *p* ≤ 0.0001). Data are shown as mean ± s.d.; *n* ≥ 3 per group. We attempted to detect NETs in the vasculature of the adipose tissue but no positive signal for any of their components was spotted at any time. We also verified numbers of neutrophils present in the blood (heart puncture). Although at first more neutrophils were detected in the peripheral blood (6 h) of obese individuals, at 24 h of sepsis their numbers were lower than those found in lean animals (Figure 3B). Thus, at the latter time point the pattern was the same as in liver sinusoids (Figure 2B). Importantly, we verified that also at 6 h of endotoxemia neutrophils of obese individuals were releasing less NETs than those of lean mice despite higher neutrophil counts (Supplementary Figure S6).

Weaker infiltration of liver sinusoids by neutrophils in obese mice than in lean ones after induction of sepsis (Figure 1 and Figure 2) was unexpected since this is one of the highlights of bacterial sepsis as demonstrated by us and others applying intravital microscopy [6,7,8,9]. Moreover, neutrophil numbers are known to be increased in the intra-abdominal adipose tissue at least at the early stages of obesity development [43,44]. For this we studied neutrophil presence in various body compartments. In the vasculature of the visceral adipose tissue we observed more neutrophils at various stages of diapedesis (rolling/adhering) in obese than lean ND mice at 24 h of sepsis confirming ongoing adipose tissue inflammation (Figure 3A and Supplementary Video S3).

In peritoneal cavity, at first (6 h), less neutrophils were present in obese mice, but the difference was not observed by 24 h of sepsis (Figure 3C). Moreover, weaker NE activity was detected in obese animals at 6 h and there was a tendency to increased neutrophil influx by late sepsis (Figure 3D). Overall, the data indicate that in the course of endotoxemia more neutrophils infiltrate peripheral tissues of obese individuals than lean ones which might result in their lower counts in systemic and liver circulation. 

### 3.5. Involvement of CXCR2, Leptin, and IL-33 in Neutrophil Influx and Casting of NETs

**Figure 4:**
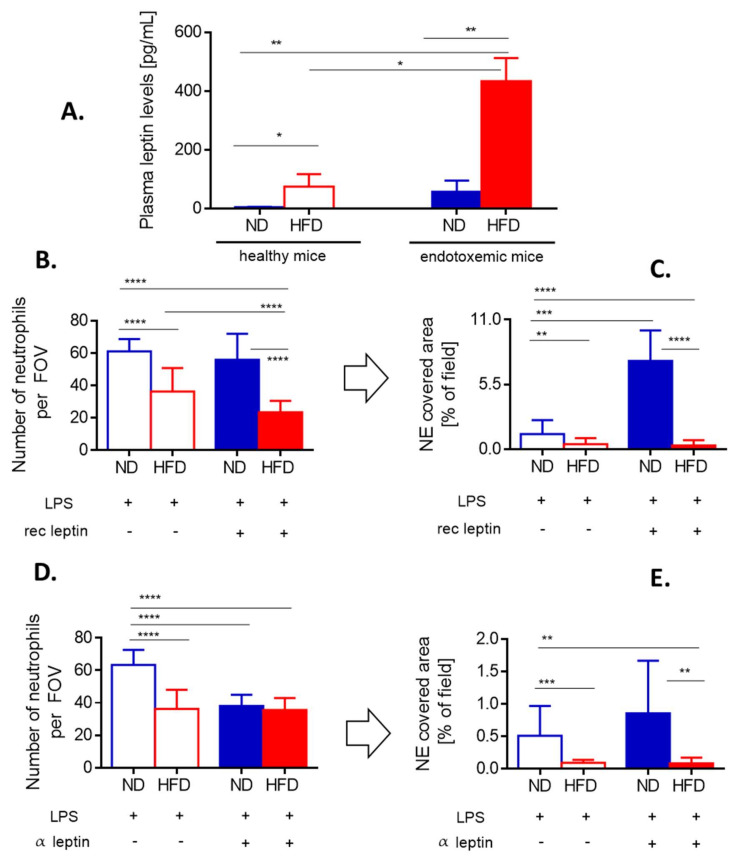
Leptin levels and impact of either exogenous leptin or endogenous leptin neutralization on neutrophil accumulation and neutrophil extracellular trap (NET) formation in liver sinusoids of lean (ND) and obese (HFD) mice. (**A**) Levels of leptin were compared between untreated (healthy) ND and HFD animals as well as endotoxemic mice, 24 h post lipopolysaccharide (LPS) injection. Some animals received a bolus of exogenous recombinant leptin (rec leptin) prior to endotoxemia induction, and subsequently neutrophil infiltration (**B**) and NET formation (**C**) in the liver vasculature were estimated. Control mice received saline. Another group of mice received mouse anti-Leptin/OB antibody to neutralize their endogenous leptin (α leptin) whereas respective controls were injected with an isotype control as detailed in Section 2.4. Upon induction of endotoxemia, neutrophil accumulation (**D**) and NET formation (**E**) in liver sinusoids were studied. Asterisks indicate significant differences between ND and HFD mice using unpaired two-tailed Student’s *t*-test (* *p* ≤ 0.05, ** *p* ≤ 0.01, *** *p* ≤ 0.001, **** *p* ≤ 0.0001). Data are shown as mean ± s.d.; *n* ≥ 3 per group.

In mice CXCL1 (KC) and CXCL2 (MIP-2), acting via CXCR2, regulate neutrophil chemotaxis and their levels are enhanced in obese individuals [45,46]. We evaluated the dependence of neutrophil infiltration into liver sinusoids on CXCR2 by pretreating obese and lean mice with its antagonist. Whereas in ND animals this profoundly decreased neutrophil infiltration, in obese mice the effect was minor (Figure 3E). However, in both groups such treatment significantly limited NET formation (Figure 3F). The same was observed when IL-33, the cytokine favoring neutrophil CXCR-2-dependent infiltration during sepsis [47], was blocked by a neutralizing antibody (data not shown). Furthermore, leptin was shown to directly correlate with IL-33 levels in mice and men [48]. Its levels were significantly higher in HFD mice, with or without endotoxemia, in comparison to their lean littermates (Figure 4A).

When HFD mice were injected with recombinant leptin, it decreased neutrophil accumulation in the liver (Figure 4B) whereas NET release was unaffected being already negligible before the treatment (Figure 4C). In contrast, in ND animals leptin profoundly activated the release of large quantities of NETs (an increase by 3–4-fold was observed) as neutrophil numbers did not increase (Figure 4B,C). Thus, we subsequently neutralized endogenous leptin and observed that this treatment inhibited neutrophil influx into the liver but only in lean mice (Figure 4D). In neither group of mice, the leptin neutralization affected NET formation (Figure 4E).

### 3.6. Weaker NET Formation by Neutrophils of Obese Mice Does Not Result from Intrinsic Defects

**Figure 5:**
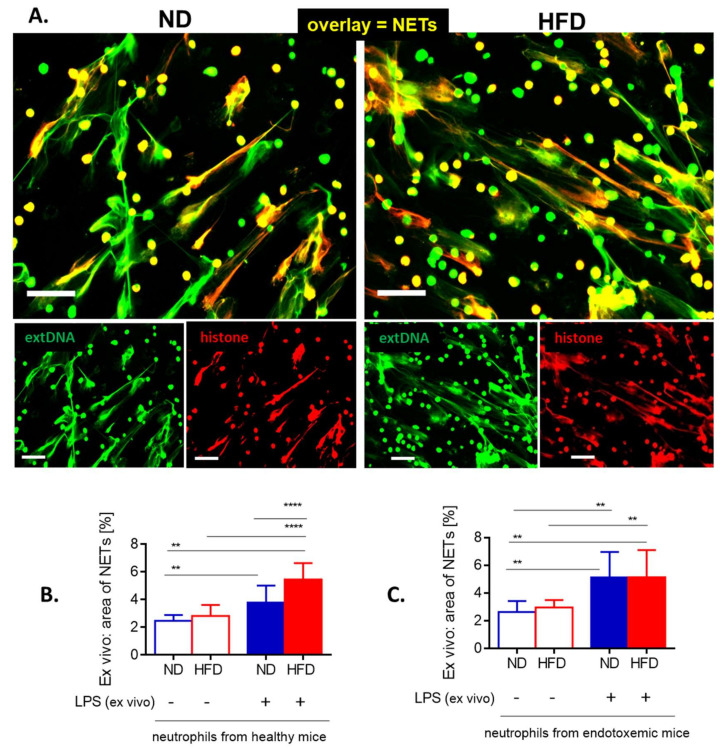
Release of neutrophil extracellular traps (NETs) by isolated bone marrow neutrophils of lean (ND) and obese (HFD) mice. (**A**) Representative images: NETs were visualized by costaining of citrullinated histone H3 (citH3, red) and extracellular DNA (extDNA, green) and their formation was evaluated 6 h after stimulation with lipopolysaccharide (LPS). The scale bar indicates 50 μm. (**B, C**) NET quantification: area (%) covered by extDNA. Neutrophils were collected either from healthy mice **(B)** or mice with LPS-induced endotoxemia **(C**). Asterisks indicate significant differences using unpaired two-tailed Student’s *t*-test (** *p* ≤ 0.01, **** *p* ≤ 0.0001). Data are shown as mean ± s.d.; *n* ≥ 3 per group.

In order to verify if the impaired NET formation by neutrophils of obese animals is intrinsic, the cells were isolated and stimulated with LPS ex vivo. Evaluation of NE (not shown), citH3 (Figure 5A) and extDNA (Figure 5A–C) revealed that neutrophils isolated from obese mice formed the same numbers of NETs than those of lean animals, or even more of them. The latter was observed when neutrophils were isolated from healthy mice (Figure 5B). 

### 3.7. Interactions of Platelets with Neutrophils and Kupffer Cells

**Figure 6:**
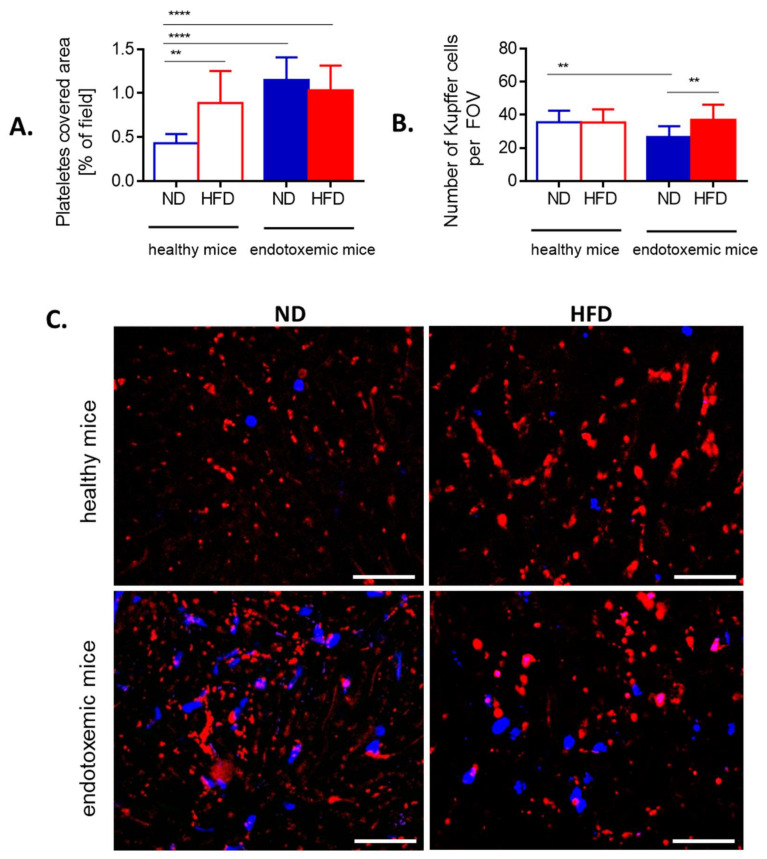
Presence of platelets and Kupffer cells in liver sinusoids of lean (ND) and obese (HFD) mice. (**A**) Area covered by platelets is expressed as percentage of covered area (as calculated by ImageJ v1.53a), and (**B**) Kupffer cells were counted per field of view (FOV) on acquired images. (**C**) Representative images of area covered by platelets (red) and localization of neutrophils (blue). The scale bar indicates 50 μm. Asterisks indicate significant differences using unpaired two-tailed Student’s *t*-test (** *p* ≤ 0.01, **** *p* ≤ 0.0001). Data are shown as mean ± s.d.; *n* ≥ 3 per group.

**Figure 7:**
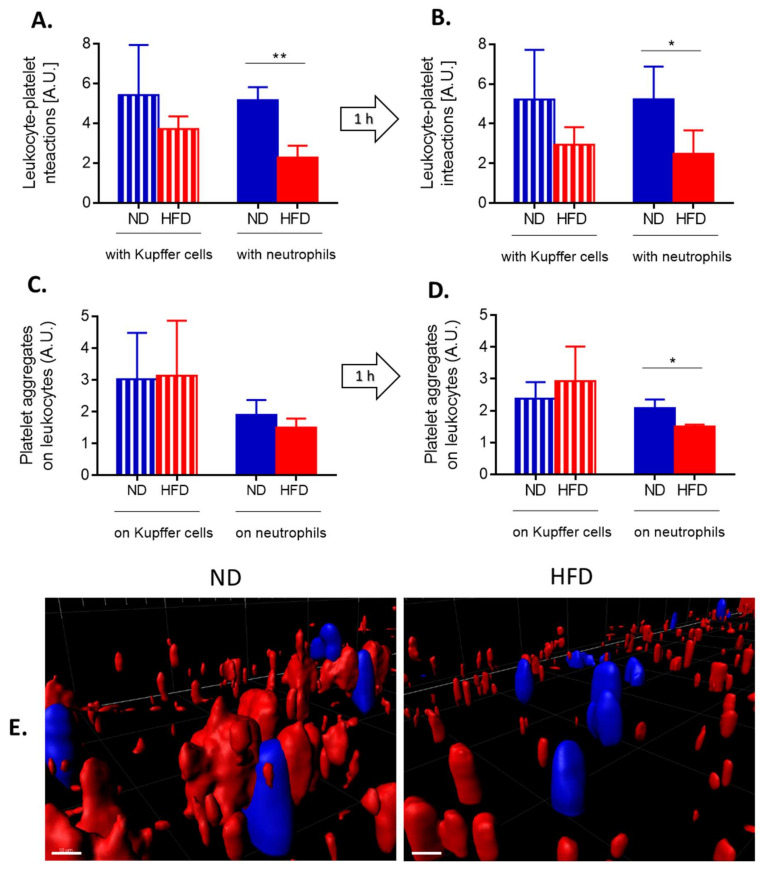
Quantification of interactions between platelets and neutrophils/Kupffer cells, and formation of platelet aggregates on the leukocytes of lean (ND) and obese (HFD) mice. Interactions between respective cells (**A** platelet–neutrophil, **B** platelet–Kupffer cells) were estimated in liver sinusoids as a sustained contact lasting at least 6 s. An average number of platelet-leukocyte interactions over 60 s is presented on the graph. Average number of platelet aggregates forming on the surface of neutrophils (**C**) and Kupffer cells (**D**) within 60 s. (**E**) Exemplary images of z stacks made through the liver of ND and HFD mice visualizing platelet aggregates of platelets (red) on neutrophils (blue). The scale bar indicates 10 μm. Asterisks indicate significant differences using unpaired two-tailed Student’s *t*-test (* *p* ≤ 0.05, ** *p* ≤ 0.01). Data are shown as mean ± s.d.; *n* ≥ 3 per group.

As release of NETs under sheer flow depends on interactions with platelets when LPS is used as an inducer [49], we tested neutrophil–platelet interactions within liver sinusoids (Figure 6 and Figure 7). Additionally, we evaluated numbers/interactions of/with Kupffer cells known to cooperate with both platelets and neutrophils present in liver sinusoids of healthy mice at early stages of systemic inflammation [39]. Obese mice had more platelets present in liver sinusoids prior to endotoxemia, but their numbers did not increase further during the course of LPS-induced inflammation unlike in their lean controls (Figure 6A,C). Additionally, in the course of MRSA-induced sepsis platelet numbers were even lower in HFD mice (Supplementary Figure S7C).

In contrast, counts of KCs were similar between ND and HFD animals although their numbers were slightly lower in lean mice during sepsis (Figure 6B). To follow interactions of platelets with KCs and neutrophils in real time, mice were subjected to IVM and when LPS was administrated via the jugular vein, their interactions were followed in real time for 1 h (Figure 7 and Supplementary Video S4). 

At the steady state, in HFD mice less platelets interacted with either phagocyte population (Figure 7A) and the same pattern was observed after 1 h (Figure 7B). However, the difference was statistically significant only in the case of platelet–neutrophil interactions. Concurrently we followed formation of platelet aggregates on the surface of KCs and neutrophils (Figure 7C). After 1 h of endotoxemia there were significantly less platelet aggregates on neutrophils but not on KCs (Figure 7D,E). However, platelet aggregates independent of either neutrophils or KCs did form in HFD animals (Figure 7E, Supplementary Video S4).

### 3.8. Transfer of Platelets Confirms Dysregulation of Platelet–Neutrophil Interactions in Obese Mice

**Figure 8:**
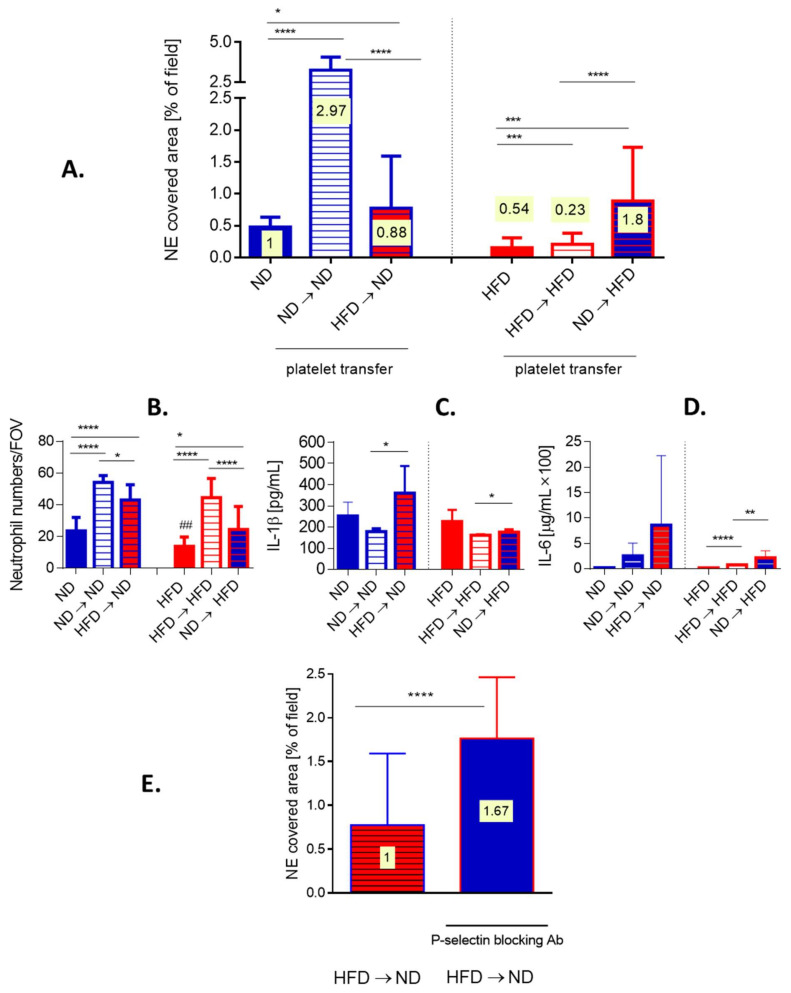
Effects of platelet transfer between lean (ND) and obese (HFD) mice on the formation of neutrophil extracellular traps (NETs), cytokine release and neutrophil accumulation in liver sinusoids. Platelets isolated from blood collected from healthy ND and HFD animals were subsequently intravenously (i.v.) injected into different recipients. Namely, platelets isolated from lean mice were injected into lean mice (ND → ND), platelets isolated from lean mice were injected into obese mice (ND → HFD), platelets isolated from obese mice were injected into lean mice (HFD → ND), and platelets isolated from obese mice were injected into obese mice (HFD → HFD). (**A**) neutrophil extracellular trap (NET) deposition in liver sinusoids and (**B**) neutrophil accumulation therein was estimated with intravital microscopy imaging (IVM), and IL-1b (**C**) and IL-6 (**D**) release into blood plasma by ELISAs. (**E**) Additionally, before transfer of platelets isolated from obese mice into lean mice (HFD → ND) they were incubated with P-selectin blocking antibody and NET formation was estimated with IVM. On bars in (**A**) and (**E**) ratio of NET-to-neutrophils is plotted (numbers on yellow background). Asterisks indicate significant differences between ND and HFD mice using unpaired two-tailed Student’s *t*-test (* *p* ≤ 0.05, ** *p* ≤ 0.01, *** *p* ≤ 0.001, **** *p* ≤ 0.0001). Data are shown as mean ± s.d.; *n* ≥ 3 per group.

To verify if indeed these are platelets of obese mice that are affecting neutrophil capacity to form NETs in vivo, we performed various transfers of platelets between obese and lean mice after which we induced endotoxemia and followed NET release. The transfer of platelets isolated from lean mice into animals on the same diet (ND → ND), significantly enhanced NET formation whereas when such mice received platelets isolated from obese mice (HFD → ND) only a minor increased in NETs was observed (Figure 8A, left).

On the other hand, when obese mice served as platelet donors, the transfer from one obese to another obese mouse (HFD → HFD) slightly improved the trap release, however, it was still weaker than in lean (ND) mice with endogenous platelet population only (Figure 8A, right). Interestingly, platelets from lean mice (ND → HFD) reconstituted ability to release NETs by neutrophils of obese animals much stronger, even beyond levels detected in ND mice (Figure 8A right versus left). The transfer of platelets itself had an impact on the milieu as evaluated by neutrophil counts and cytokine levels. In particular, either platelet transfer induced the influx of neutrophils into liver sinusoids (Figure 8B). Moreover, transfer of HFD-derived platelets increased the release of IL-1b and IL-6 in lean recipients (HFD → ND) (Figure 8C,D). Receiving exogenous platelets did not stimulate IL-1b in obese mice but slightly increased IL-6 levels independently of the donor’s origin (Figure 8C,D). The cytokine data reveals that indeed, HFD-derived platelets carry an altered phenotype. To make sure that the increase in the capacity to induce NET upon platelet transfer is not due to additional neutrophils, NET-to-neutrophil ratio was estimated (Figure 8A,E, numbers plotted on bars). These data corroborate previous conclusions, and in particular that transfer of ND platelets did induce stronger NET formation than that of HFD platelets. In order to verify if the difference in ability of platelets to activate neutrophils to cast NETs results from altered integrin expression on the latter cells, we evaluated expression of LFA-1 (b2(CD18)/aL(CD11a)) (Supplementary Figure S8A,B). The integrin was shown previously to be critical for NET release by neutrophils infiltrating liver sinusoids [9]. However, we did not detect any differences in LFA-1 expression on neutrophils isolated from ND or HFD mice or animals which received transferred platelets (ND → HFD or HFD → ND) (Supplementary Figure S8B). We also excluded contamination of platelets used in transfer experiments with either neutrophils or monocytes/macrophages (Supplementary Figure S9). Therefore, in following experiments we focused on platelets. As CD62P expression is enhanced on platelets of obese mice ([50] and our unpublished data) we blocked this selectin on them prior to their transfer to lean animals (HFD → ND). Such treatment significantly increased neutrophil capacity to cast NETs, per se and when recalculated to the NET-to-neutrophils ratio (1:1.67; Figure 8E and numbers plotted on bars).

## 4. Discussion

Inflammatory host responses to infection can lead to sepsis and lethal septic shock if overactivated and/or dysregulated [1,2,3,51]. Additionally, endotoxins are found in the blood of patients with sepsis and their presence is associated with shock and multiple organ dysfunction [52]. If indeed sepsis outcome is more favorable in obese individuals, their immune response might be different, and in particular weaker, than in lean individuals. In line with this, herein we show that both neutrophil infiltration and NET formation in liver sinusoids are less pronounced in obese mice than in their counterparts with moderate weight.

This effect is independent of the obesity type be it induced by high fat diet (more representative of human obesity) or leptin mutation (a rare cause of obesity in humans [53]). It is also independent of the inducing agent, Gram^−^ vs. Gram^+^ bacteria/bacterial components. One of the highlights of sepsis is organ(s) failure and mostly the respiratory, renal, neural, cardiovascular, hematopoietic/hematological, and hepatic systems are affected [51]. In animal models, the injury was often connected to NET formation, and namely to the thrombogenic or cytotoxic action of NET components [6,9,54,55], but most importantly, NET involvement was also confirmed to accompany human sepsis pathology [55,56,57]. 

In our studies, livers of obese mice were found damaged even prior to sepsis, especially in ob/ob mice, yet they were surprisingly not further damaged in the course of endotoxemia as observed in lean mice. Obese animals were also losing less weight (recalculated as its % to correct for differences in body weight) upon induction of endotoxemia whereas a significant, but transient, weight loss is one of the highlights of murine sepsis [58]. The lack of further liver injury might be a consequence of significantly weaker NET casting by neutrophils of obese mice. In fact, to our surprise, neutrophils were less numerous in livers of obese animals, especially those on HFD, not only during sepsis but also even in untreated animals while we usually connect the state of obesity with low grade inflammation [30,42]. However, when imaging vasculature of the adipose tissue of obese mice we indeed detected numerous neutrophils at various stages of diapedesis whereas in lean animals only single neutrophils either adhered or rolled on the endothelium. Additionally, in the peritoneal cavity (as an exemplary peripheral compartment) neutrophils started to gradually gather over time after sepsis induction although at first (6 h) their numbers were lower in obese mice and so was NE activity in peritoneal lavage. However, in general circulation, prior to sepsis and on its early stages, neutrophils were more numerous in obese individuals. Whereas with time (after 24 h) also blood neutrophil counts dropped down below levels detected in their lean counterparts which correlates with observations made in liver sinusoids. 

Further focusing on neutrophil accumulation in the liver, in lean mice the process depended on IL-33-CXCR2 axis and leptin as to be expected during LPS-induced systemic inflammation. IL-33 controls neutrophil recruitment during sepsis by preventing downregulation of CXCR2 and inhibition of chemotaxis induced by the activation of TLR4 in mouse and human neutrophils [47]. However, blockage of CXCR2 did not prevent neutrophil accumulation in liver sinusoids of obese animals. The receptor ligands in mice are CXCL1 (KC) and CXCL2 (MIP-2), known to regulate neutrophil chemotaxis [59], and their levels are also elevated in obesity [31,45]. CXCR2 works in tandem with CXCR1 and they share numerous ligands, however, some of them are unique to the former, including KC and MIP-2 [60,61]. As the levels of the two chemokines are increased in obesity/obese mice desensitization of CXCR2 (homo- or heterologous GPCR desensitization [62]) during sepsis cannot be excluded. However, it is not likely considering that inhibition of CXCR2 did decrease NET formation in obese mice (please compare below). Another possibility being the dependence of neutrophil chemotaxis on CXCL5 (LIX), binding both CXCR1 and CXCR2 [61], which levels are also increased in obesity [63]. Interestingly, CXCL5 drives obesity to diabetes [63] and our mice on HFD started to exhibit elevated glucose levels. During early sepsis (6 h) numbers of circulating neutrophils were higher in obese (HFD) mice and this interestingly correlates with the observation that more (lean) mice survived sepsis if additional neutrophil influx was induced at its beginning by the application of KC and MIP-2 [64].

However, the most striking observation was that neutrophils of obese mice casted less NETs than those of their lean counterparts. The traps observed in obese mice were composed of all tested components, were sensitive to DNAse I (not shown) yet it was significantly less of them. This did not result from lower neutrophil numbers as revealed by NET-to-neutrophil ratio. Moreover, in ob/ob mice there were only negligible NETs present even though they had more neutrophils in liver sinusoids than HFD animals. These results were further confirmed by analyzing plasma concentration of active NE. In obesity, levels of NE in the blood are increased and this protease is pivotal in obesity development (no NE, no obesity) [42]. The inflammatory state associated with obesity originates in the expanding adipose tissue, and is mainly associated with infiltration by leukocytes, particularly M1 macrophages and T cells but also neutrophils at its onset [65] which explains elevated NE levels. However, in the herein studied obese mice, NE levels did not further increase during endotoxemia nor were they altered by the inhibition of NET formation. Thus, indicating that it was free circulating protease, and not NET-NE, which levels are known to be increased in obesity [42,44]. In contrast, in lean mice NE activity originated mostly from NETs. Overall, we clearly show that in obese mice neutrophils cast less NETs in liver sinusoids, but also indirectly that this effect is not limited to the liver. Thus far, direct measurements of NETs in obese individuals were not reported. However, the measurement of single NET proteins or application of a NET inhibitor (Cl-amidine) was shown to decrease endothelial vasodilation in the mesentery of otherwise healthy obese mice [66]. In another study, Cl-amidine did not impact adipose tissue inflammation in mice with obesity [67]. In humans, increased levels of MPO-DNA complexes circulating in the plasma were detected in obese individuals [68]. On the other hand, in the course of influenza similar numbers of NETs were detected in lung sections of obese and lean mice [69]. Therefore, the thus far reported data was inconsistent in regard to NET formation by neutrophils of obese individuals, however, direct NET evaluation was not performed till now.

Although neutrophil chemotaxis into the liver did not depend on the IL-33-CXCR2 axis, the NET formation did. KC and MIP-2, besides controlling neutrophil infiltration, can induce NET formation [70] and in our studies, neutrophils of both lean and obese mice released less NETs upon CXCR2 inhibition confirming the ligand impact. Additionally, data on leptin involvement is of interest. Levels of this “satiety hormone” directly correlate with increased NE and IL-33 concentration in obese mice and men [42,48] and leptin itself is increased in obesity and inflammation [71]. Interestingly, elevated levels of leptin have been shown to have a benefit in terms of mortality, but this result is not uniform among all studies [72]. Although it is partly controversial [73], some studies show that leptin can also act as a chemoattractant for neutrophils [74]. Whereas these studies were executed on isolated neutrophils, here we show in vivo that indeed leptin neutralization partially decreased their accumulation in liver sinusoids of lean mice. Interestingly, in these animals, additional exogenous leptin did not further increase neutrophil accumulation but it did increase NET formation. Up till now the role of leptin in NET induction is unclear but our results indicate that indeed it might be involved. However, when we neutralized the endogenous leptin, the NET formation proceeded normally, thus it is the excess of leptin that triggers NETting. The lack of responsiveness of obese mice to leptin might result from “cellular leptin resistance” characteristic to obesity [75]. However, although neither leptin manipulation affected NET formation by neutrophils of obese mice, exogenous leptin administration further inhibited neutrophil accumulation again suggesting leptin resistance/desensitization.

Neutrophils isolated from septic obese mice and stimulated ex vivo with LPS casted the same numbers of NETs as did neutrophils of lean mice, whereas those isolated from healthy animals formed even more NETs. The latter finding suggests that neutrophils of otherwise healthy obese mice carry a proinflammatory phenotype which makes them more prone for NET release, and thus, that these are neutrophil-independent conditions present in vivo that prevent neutrophils from casting larger quantities of NETs. Whereas, neutrophils isolated from septic individuals formed the same numbers of NETs independently of their origin. This might reflect on the exhausted neutrophil phenotype typical for sepsis and characterized by elevated expression of immunosuppression-associated markers (i.e., PD-L1) as well as adhesion molecules resulting in pathogenic and immune-suppressed phenotype [76]. Nevertheless, our ex vivo studies clearly show a lack of intrinsic problems with casting NETs by neutrophils of obese mice. This turned our attention to the inflammatory milieu because in vivo NET formation upon LPS inoculation was shown to depend on platelets in shear stress conditions such as in the vasculature [49]. In particular, upon detection of LPS via platelet TLR4, thrombocytes bind to neutrophils leading to their robust activation and NET release [49]. Thus, we evaluated the presence of platelets in liver vasculature and their interactions with neutrophils. Although generally more platelets were detected in obese mice in liver sinusoids prior to endotoxemia when compared to lean controls, their levels stayed the same during the inflammation whereas they did increase in lean controls. Platelet counts are increased in obesity [77] and this was also confirmed in animal models [78]. Furthermore, platelet functioning is dysregulated (altered adhesion, hyperactivation) overall favoring a mild-to-moderate hypercoagulable state. On the other hand, sepsis is characterized by persistent thrombocytopenia [21]. Its causes are not clear but most probably the phenomenon results from reduced platelet production, enhanced turnover, or spontaneous aggregation. Indeed, animal models show that upon administration of LPS there is a drop in the circulating platelet count, and concomitantly platelets form large aggregates in the liver sinusoids (IVM) [22]. Furthermore, platelets interact primarily with neutrophils already adherent within the liver, and the platelet aggregates are the same size as, or larger than, the adherent neutrophils [22]. When studying the course of sepsis in obese individuals (the Western diet), increased platelet adhesion was also observed during polymicrobial sepsis [79]. In general, in obese individuals or mice with sepsis exaggerated thrombogenic responses are observed [79,80,81]. In line with this, with IVM we observed more spontaneous platelet aggregates in sinusoids of obese mice, healthy or with endotoxemia. However, we detected less platelets interacting with neutrophils during endotoxemia in obese mice and consequently less platelet aggregates on neutrophils were deposited in contrast to Kupffer cells (KCs). Platelet–KC interactions are known to operate at the early stages of bacterial sepsis [39]. Namely, under basal conditions, platelets form transient ‘touch-and-go’ interactions with KCs which transit to sustained adhesion on the KC surface to encase the foreign body. The latter interactions depend on von Willebrand factor (VWF) on KCs. Levels of VWF are increased in obesity and they result from both an increased synthesis and impaired clearance [82] rather than from shedding which explains an unaltered formation of KC-platelet aggregates.

As the platelet–neutrophil interactions were diminished, we evaluated expression of selected integrins (LFA-1) on neutrophils but no differences were detected between lean and obese mice indicating that the problem is rather related to platelets. Thus, subsequently we performed a platelet transfer between HFD and ND healthy mice prior to the induction of endotoxemia. Transfer of additional platelets between ND donor/ND recipient significantly increased casting of NETs but most importantly, transfer of ND platelets to a HFD recipient significantly improved NET formation. These results were corrected for numbers of neutrophils (NET-to-neutrophil ratio) that infiltrated liver sinusoids in response to platelet injection. However, transfusion of platelets originating from HFD mice did not affect NETting in either HFD nor ND animals clearly demonstrating that these platelets are dysfunctional. Scrutinizing for the underlying cause we blocked expression of P-selectin (CD62P) on these platelets prior to their transfer knowing that expression of this selectin is increased on platelets of obese individuals ([50] and our unpublished data). This treatment increased the capacity of HFD platelets to induce NET release by neutrophils indicating that the impaired NET formation in obese septic mice is related to P-selectin overexpression. Thus, our hypothesis is as follows, overactivated platelets of obese mice form spontaneous aggregates preferably with each other rather than with neutrophils limiting platelet–neutrophil interactions required for NET release. In some studies, it was shown that blocking P-selectin prevents their binding to its ligand (P-selectin glycoprotein ligand-1, PSGL-1) [83] expressed also on platelets [84] and so their aggregation. Therefore, we hypothesize that when overexpressed P-selectin is neutralized, the platelet-platelet binding is limited, and their higher number is available, so NET formation is favored. However, this hypothesis was not tested thus far and, furthermore, we do not know if the actual platelet–neutrophil interactions leading to the release of NETs involve P-selectin itself or rather other platelet receptors. The mechanism of platelet dysfunctionality during sepsis in obese mice still needs to be detailed and requires further studies.

The presented data shows that during systemic inflammation the immune response differs between individuals with regular/lean body weight and obesity. Despite an increased inflammatory state in the adipose tissue, in the liver, recognized nowadays as an important immunological organ [85], the inflammatory reaction is weaker and not only because of lower neutrophil infiltration but foremost because of reduced formation of platelet aggregates on neutrophils leading to diminished NET release. Whereas, the two latter processes induce hepatic dysfunction in early sepsis [86] and restoration of liver function lowers morbidity and mortality rates in patients with systemic inflammation [21]. As we have also linked lower NET formation accompanying obesity with an impaired expression of P-selectin, we propose dysfunctionality of platelets as an important factor behind the obesity paradox in sepsis. Hyperactivation of thrombocytes is well recognized as an increased risk factor in patients with systemic inflammation [21] and so is NET formation [87]. Therefore, inhibition of these two processes might lead to improved outcome in sepsis-treatment.

## Data Availability

All data is contained within the manuscript and the supplement.

## Supplementary Materials

The following are available online at https://www.mdpi.com/2073-4409/10/2/384/s1, Figure S1: Body weight, food and water intake by lean (ND) and obese (HFD) mice, Figure S2: Metabolic parameters of lean (ND) and obese (HFD) mice, Figure S3: Impact of obesity on organ weight and liver morphology and injury, Figure S4: Deposition of neutrophil extracellular traps (NETs) in liver sinusoids of healthy mice and during endotoxemia (24 h) in wild-type (wt, left) and ob/ob (right) mice as well as high fat diet-fed (HFD) animals and their control littermates, Figure S5: Kinetics of NET formation and neutrophil accumulation in liver sinusoids during endotoxemia in lean and obese mice, Figure S6: Comparison of NET formation between different time points of endotoxemia, and detection of various NET components (neutrophil elastase, NE versus H2AX) in liver sinusoids of lean (ND) and obese (HFD) mice), Figure S7: Comparison of NET formation and platelet accumulation in liver sinusoids at 24 h of Methicillin-resistant Staphylococcus aureus (MRSA)-induced sepsis in lean (ND) and obese (HFD) mice versus LPS inoculation (endotoxemia), Figure S8: Expression of LFA-1 on neutrophils from lean (ND) and obese (HFD) mice and animals subjected to platelet transfer, Figure S9: Representative data on verification of platelet purity in transfer experiments, Video S1 Neutrophil extracellular trap (NET) formation in liver sinusoids of lean (ND) and obese (HFD) mice during endotoxemia (24 h post i.p. LPS), Video S2 Neutrophil extracellular trap (NET) formation in liver sinusoids of lean (wt) and obese (ob/ob) mice during endotoxemia (24 h post i.p. LPS), Video S3 Neutrophils in the vasculature of the adipose tissue of lean (ND) and obese (HFD) mice, Video S4 Interactions of platelets with neutrophils in liver sinusoids as captured with IVM.
