# Supplementary material for: Scrutinizing Mechanisms of the ‘Obesity Paradox in Sepsis’: Obesity Is Accompanied by Diminished Formation of Neutrophil Extracellular Traps (NETs) Due to Restricted Neutrophil–Platelet Interactions
Source: Cells. 2021 Feb 12;10(2):384. doi: 10.3390/cells10020384 (PMC7918512; doi:10.3390/cells10020384)
Supplement: Supplementary file 1 [file cells-10-00384-s001.zip › cells-1050081 Final Supplementary files for XML/SUPPL EMENTARY cells-1050081- CORRECTIONS PROOF.docx]

Supplementary Figures


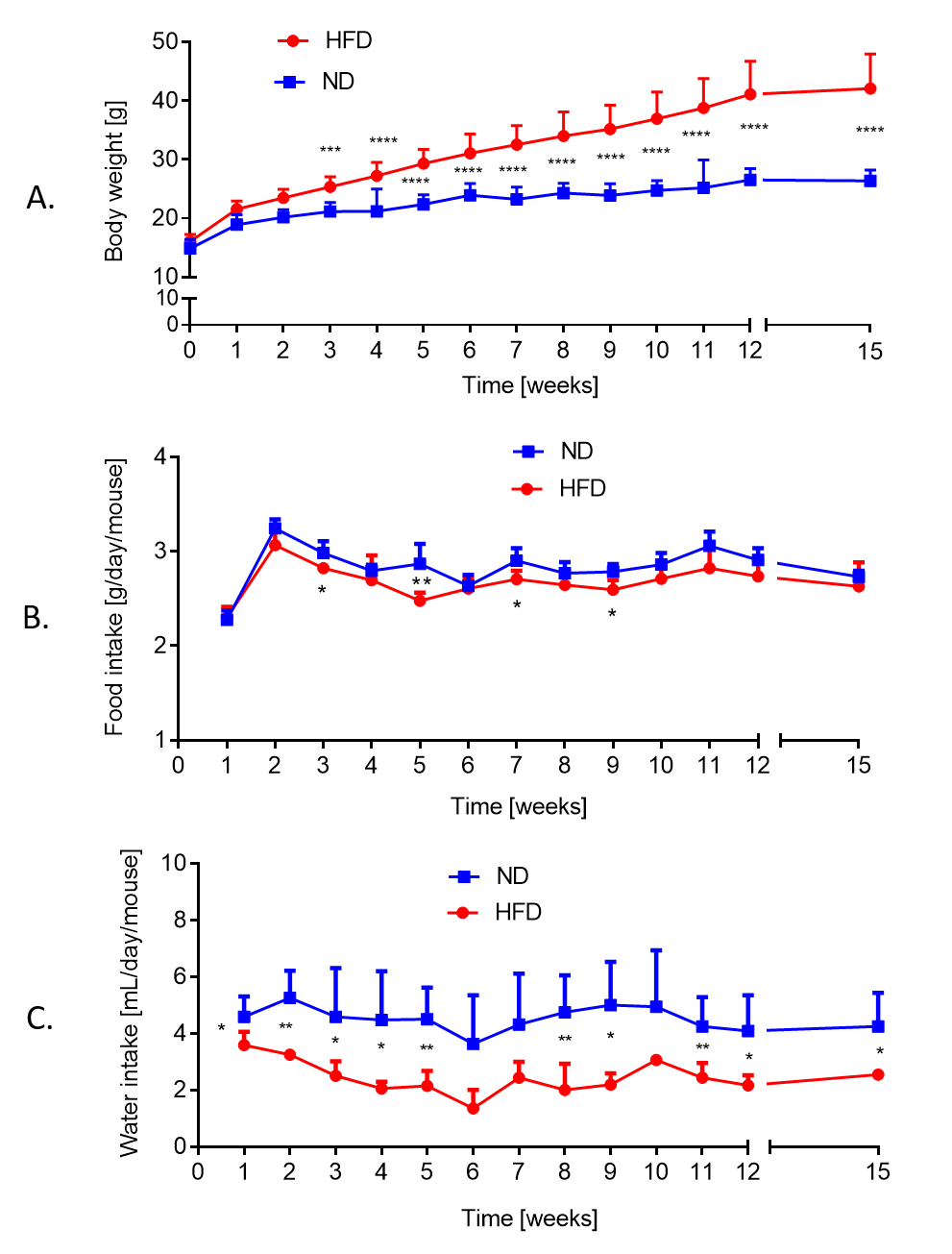


**Supplementary Figure S1.** Body weight, food and water intake by lean (ND) and obese (HFD) mice. The body weight (**A**) was monitored weekly and food (**B**) and water (**C**) intake daily (average values from each week are presented on the graph). The presented data refers to untreated mice, prior to any experimental treatments. Asterisks indicate significant differences between ND and HFD mice using unpaired two-tailed Student’s *t*-test (* *p* ≤ 0.05, ** *p* ≤ 0.01, *** *p* ≤ 0.001, **** *p* ≤ 0.0001). Data are shown as mean ± s.d.; *n* ≥ 3 per group.


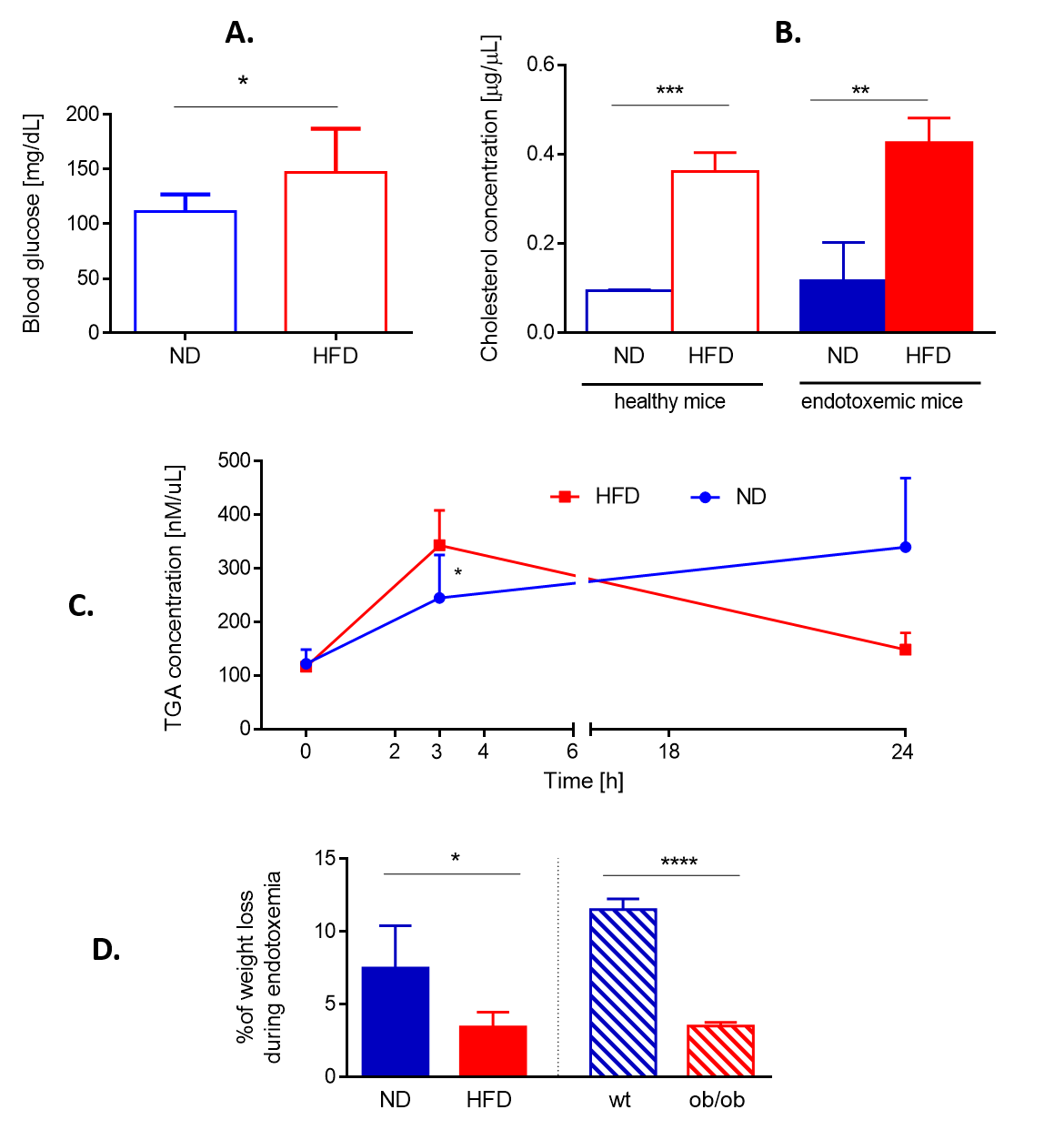


**Supplementary Figure S2.** Metabolic parameters of lean (ND) and obese (HFD) mice. (**A**) Blood glucose levels were estimated in healthy, untreated mice, and cholesterol (**B**) and triglyceride (TGA) (**C**) levels both in healthy and endotoxemic mice at 24 h and over several hours after LPS administration, respectively. Both groups of mice lost some weight in the course of endotoxemia (shown here for 24 h) and it is expressed as a percentage of weight loss (D) to normalize for differences in their body weight (Supplementary Figure 1A). Asterisks indicate significant differences between ND and HFD mice using unpaired two-tailed Student’s *t*-test (* *p* ≤ 0.05, ** *p* ≤ 0.01, *** *p* ≤ 0.001, **** *p* ≤ 0.0001). Data are shown as mean ± s.d.; *n* ≥ 3 per group.


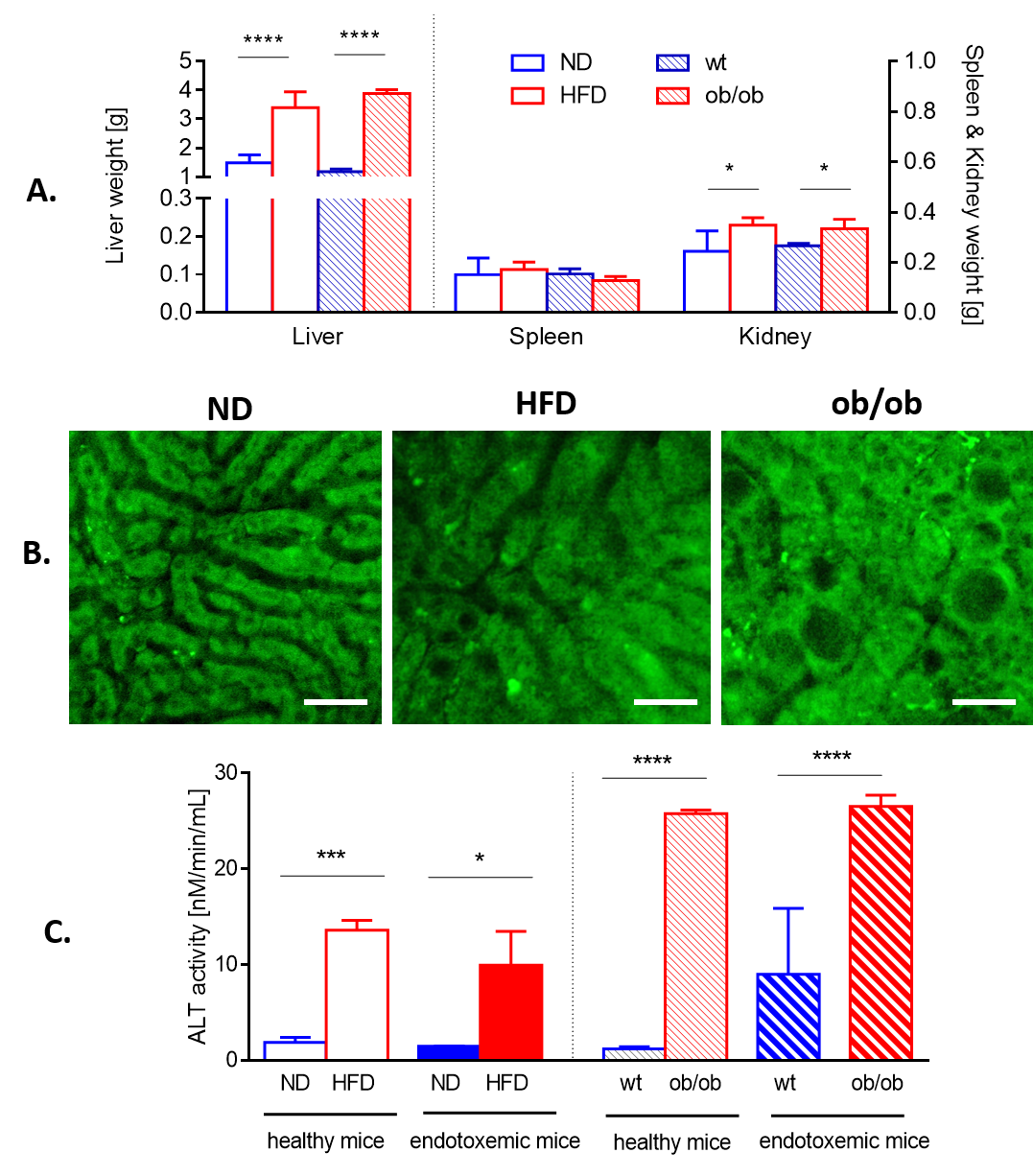


**Supplementary Figure S3.** Impact of obesity on organ weight and liver morphology and injury. (**A**) weight of livers, spleens, and kidneys of NE and HFD mice was verified upon their dissection, and liver morphology was imaged with IVM. Representative images of livers of ND HFD and ob/ob healthy mice are shown in (**B**). The scale bar indicates 50 μm. Serum alanine transaminase (ALT) levels were measured in blood plasma of lean (ND) and (HFD) animals as well as wt and ob/ob mice prior to sepsis and 24 h post LPS i.p. injection (**C**). Asterisks indicate significant differences between lean (either ND or wt) and obese (either HFD or ob/ob) mice using unpaired two-tailed Student’s *t*-test (* *p* ≤ 0.05, *** *p* ≤ 0.001, **** *p* ≤ 0.0001). Data are shown as mean ± s.d.; *n* ≥ 3 per group.


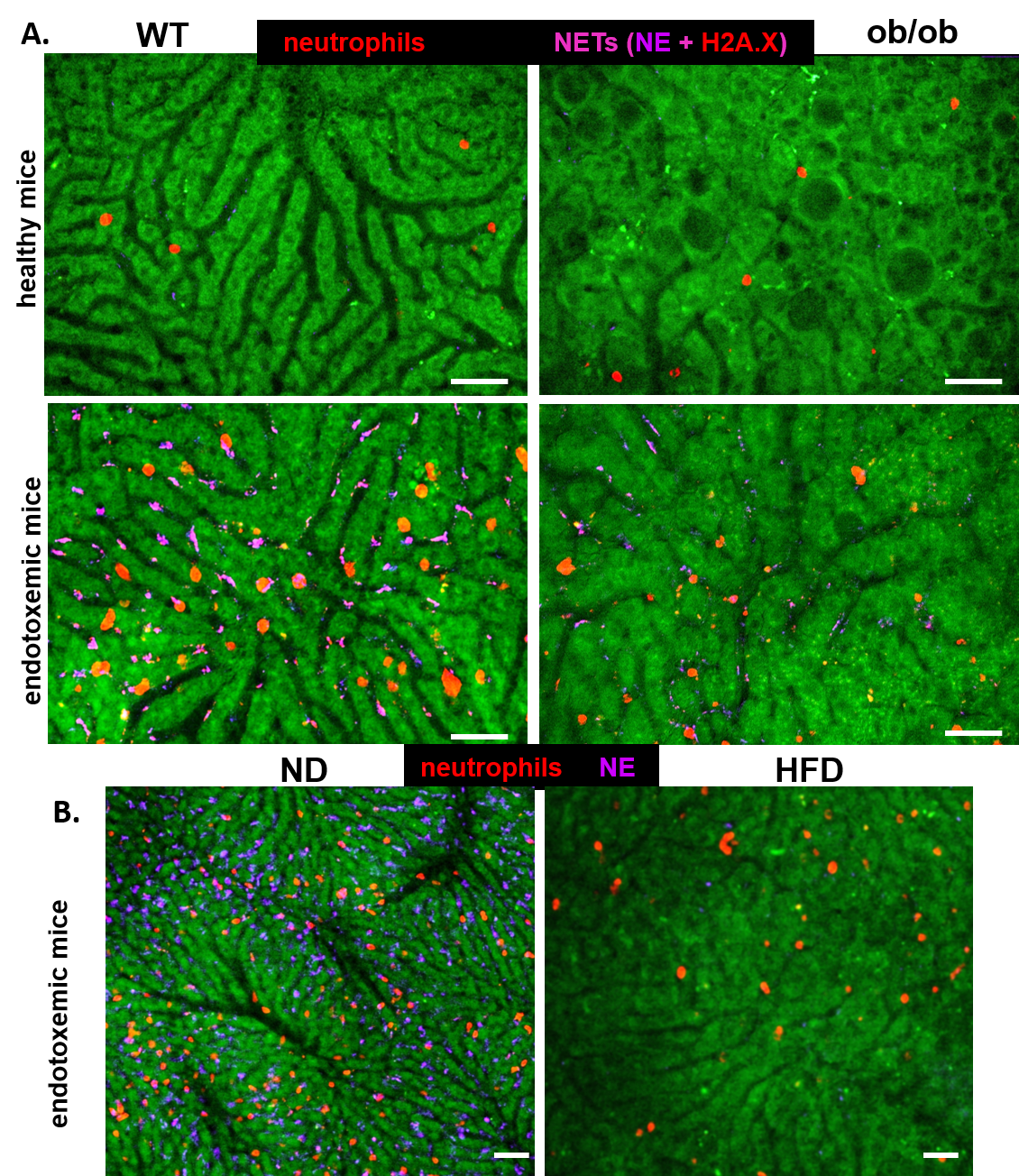


**Supplementary Figure S4.** Deposition of neutrophil extracellular traps (NETs) in liver sinusoids of healthy mice and during endotoxemia (24 h) in wild-type (wt, left) and ob/ob (right) mice as well as high fat diet-fed (HFD) animals and their control littermates. (**A**) On images autofluorescent hepatocytes (dim green) can be observed in between which sinusoids are localized (black ducts). In the latter structures neutrophil elastase (NE, violet), histone H2A.X (red), and extracellular DNA (extDNA, bright green) overlaying signal is visible lining along endothelium. (**B**) Representative overlays of NETs and neutrophils of lean (ND) and obese (HFD) mice at 24 h of endotoxemia. Magnification 20× (**A**) and 10× (**B**). The scale bar indicates 50 μm.


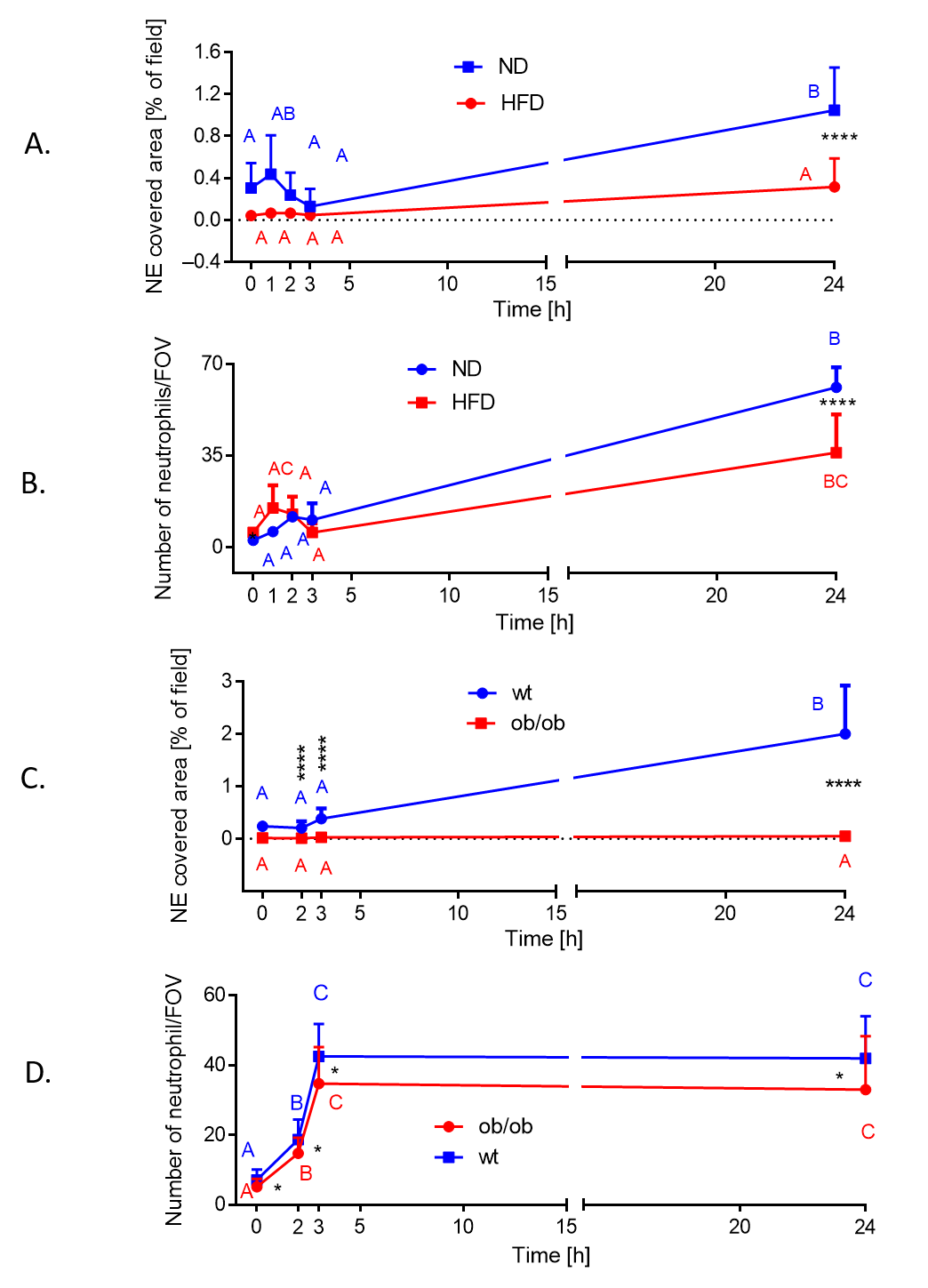


**Supplementary Figure S5.** Kinetics of NET formation and neutrophil accumulation in liver sinusoids during endotoxemia in lean and obese mice. The analyses were performed in both models of obesity, in high fat diet (HFD) animals and their controls (ND) (left column), and ob/ob mice and their wild-type counterparts (wt) (right column). (**A**) Quantitative analysis of NETs within the liver sinusoids: area (%) covered by NE in ND vs. HFD mice. (**B**) The numbers of infiltrating neutrophils in liver sinusoids of ND vs. HFD animals were quantified with ImageJ software v1.53a and are expressed as number per field of view (FOV). (**C**) Quantitative analysis of NETs within the liver sinusoids of wt vs. ob/ob mice. (**D**) The numbers of neutrophils in liver sinusoids of wt vs. ob/ob mice per field of view (FOV). Asterisks indicate significant differences between lean (either ND or wt) and obese (either HFD or ob/ob) mice using unpaired two-tailed Student’s *t*-test (* *p* ≤ 0.05, **** *p* ≤ 0.0001). Changes in time were tested by one-way ANOVA (post hoc Bonferroni) analysis (different letters indicate statistically significant differences between given time points within one group). Data are shown as mean ± s.d.; *n* ≥ 3 per group.


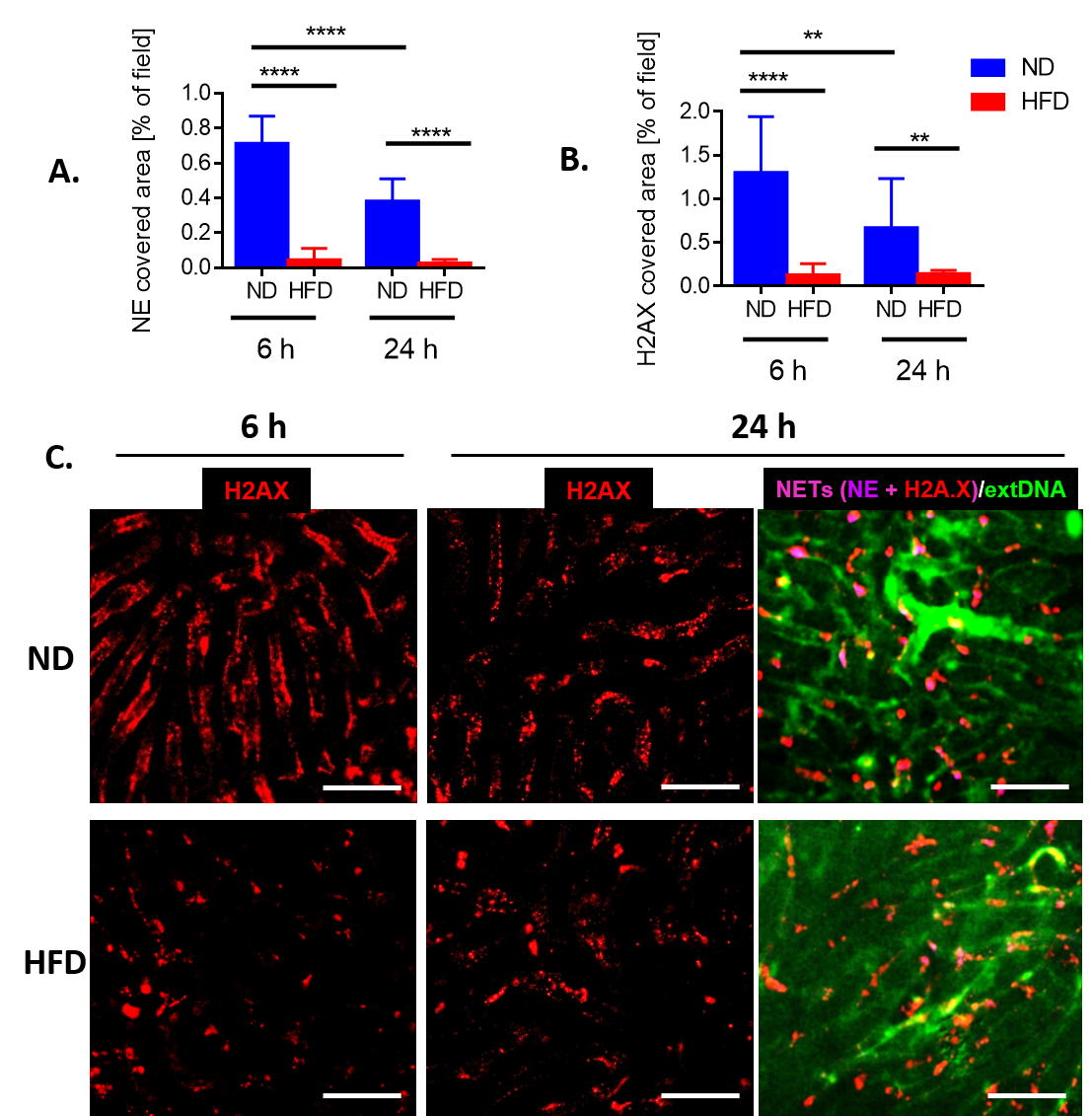


**Supplementary Figure S6.** Comparison of NET formation between different time points of endotoxemia, and detection of various NET components (neutrophil elastase, NE versus histone, H2A.X) in liver sinusoids of lean (ND) and obese (HFD) mice. Quantitative analysis of NET-NE (**A**) and NET-H2AX (**B**) in sinusoids (% of area covered by each signal). (**C**) Representative images of NET-H2AX (red) were acquired with SD-IVM. Overlay of NE (violet)-H2AX (red)-extDNA (bright green) signals indicative of NETs is shown in the right panel. The scale bar indicates 50 μm. Asterisks indicate significant differences between lean and obese mice using unpaired two-tailed Student’s *t*-test (** *p* ≤ 0.01, **** *p* ≤ 0.0001). Data are shown as mean ± s.d.; *n* ≥ 3 per group.


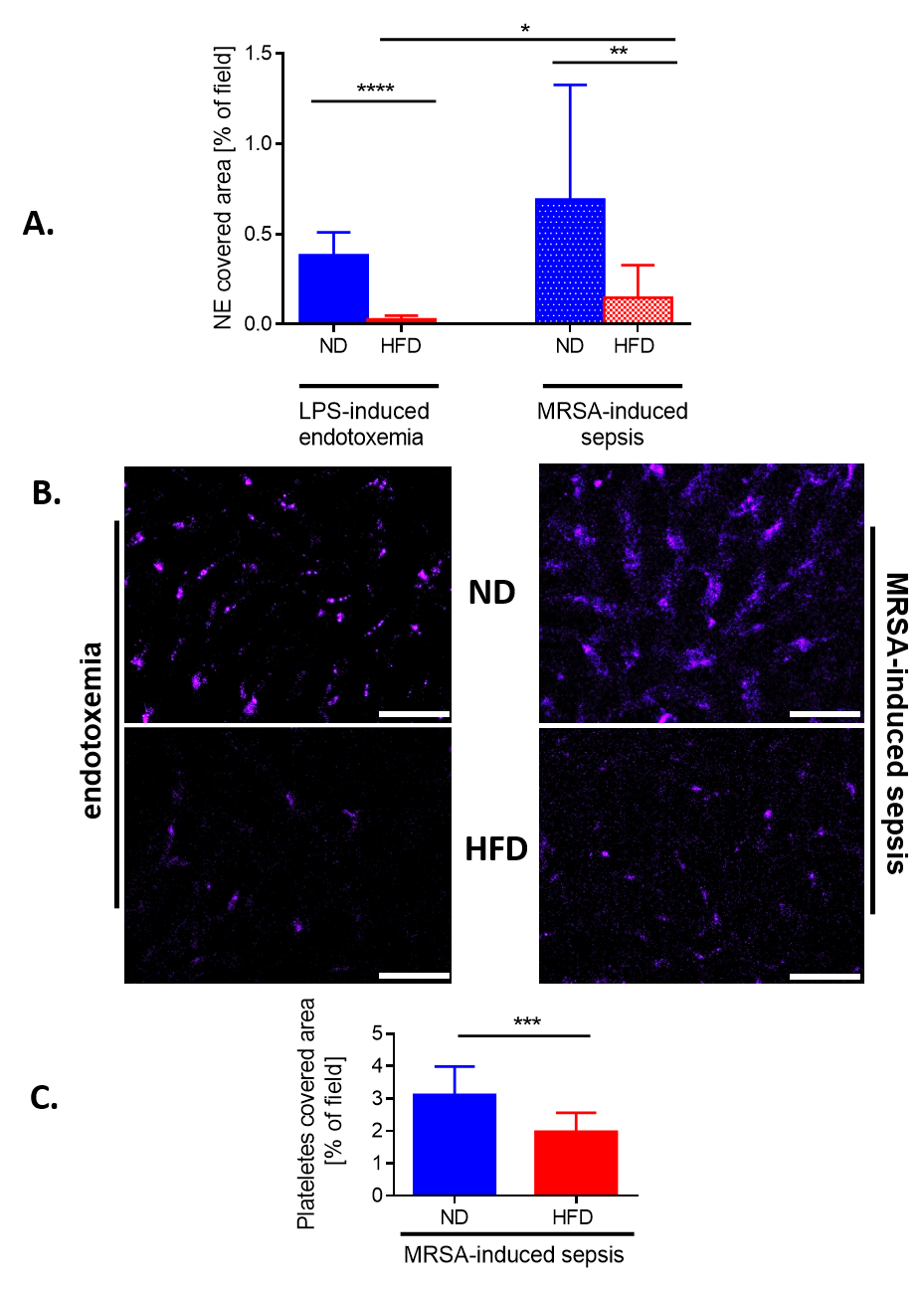


**Supplementary Figure S7.** Comparison of NET formation and platelet accumulation in liver sinusoids at 24 h of Methicillin-resistant *Staphylococcus aureus* (MRSA)-induced sepsis versus LPS inoculation (endotoxemia) in lean (ND) and obese (HFD) mice. (**A**) Quantitative analysis of NETs within the liver sinusoids: area (%) covered by neutrophil elastase (NE, violet) in MRSA and LPS-treated animals. (**B**) Representative images of NETs were acquired with SD-IVM. The scale bar indicates 50 μm. (**C**) Area covered by platelets in mice with MRSA-induced sepsis is expressed as percentage of covered area (as calculated by ImageJ). Asterisks indicate significant differences between lean and obese mice using unpaired two-tailed Student’s *t*-test (* *p* ≤ 0.05, ** *p* ≤ 0.01, *** *p* ≤ 0.001, **** *p* ≤ 0.0001). Data are shown as mean ± s.d.; *n* ≥ 3 per group.


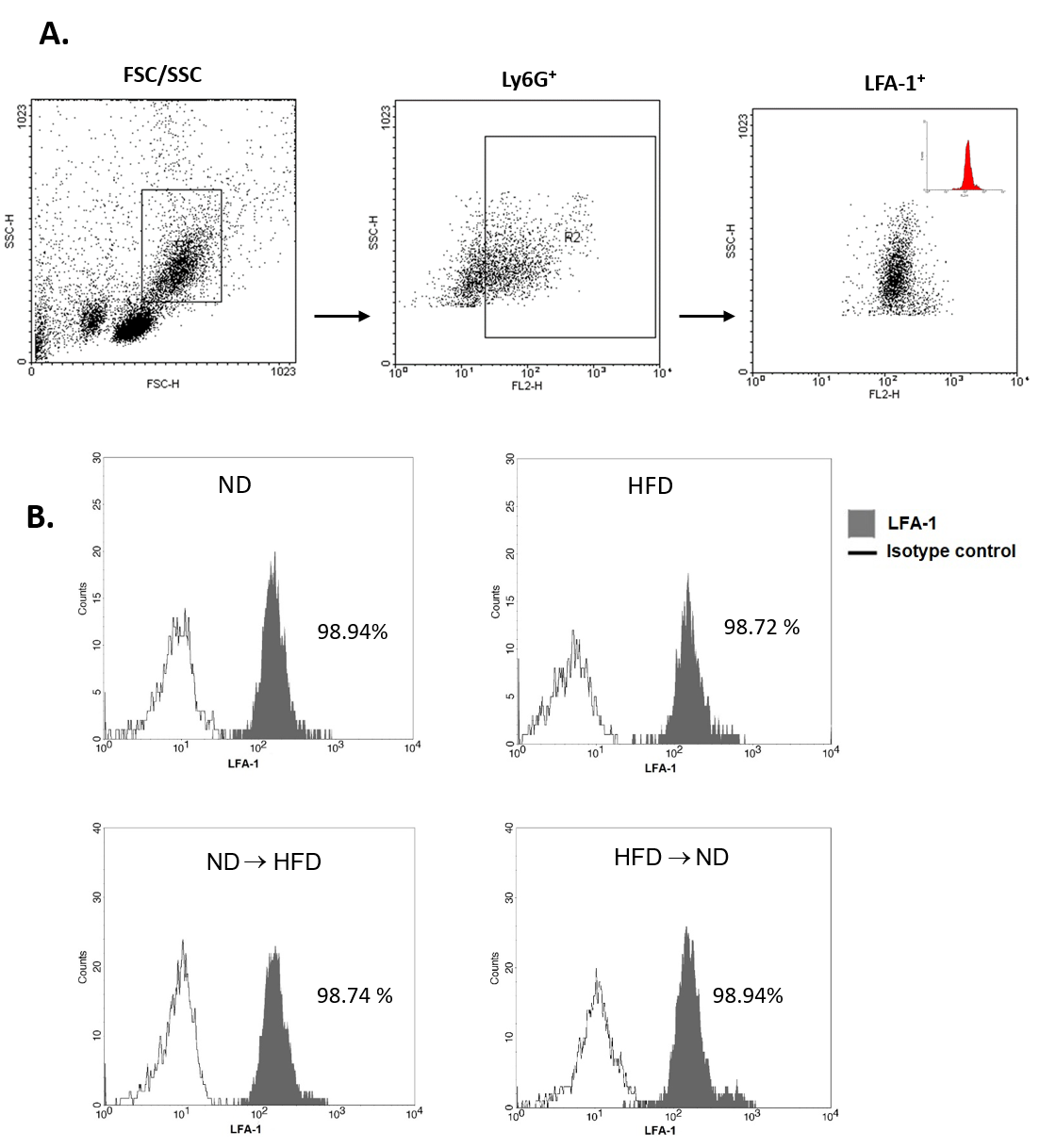


**Supplementary Figure S8.** Expression of LFA-1 on neutrophils from lean (ND) and obese (HFD) mice and animals subjected to platelet transfer. Panel (**A**) shows the gating strategy—within large granulated cells (R1), Ly6G^+^ neutrophils were gated (R2) on which LFA-1 expression was evaluated. (**B**) Representative flow cytometry histograms of Ly6G-positive neutrophils in each group are shown. The number of Ly6G^+^ cells was always >98%. Lean mice (ND), animals on high-fat diet (HFD), HFD mice that received platelet transfer from lean mice (ND →HFD), ND mice that received platelet transfer from obese mice (HFD → ND).


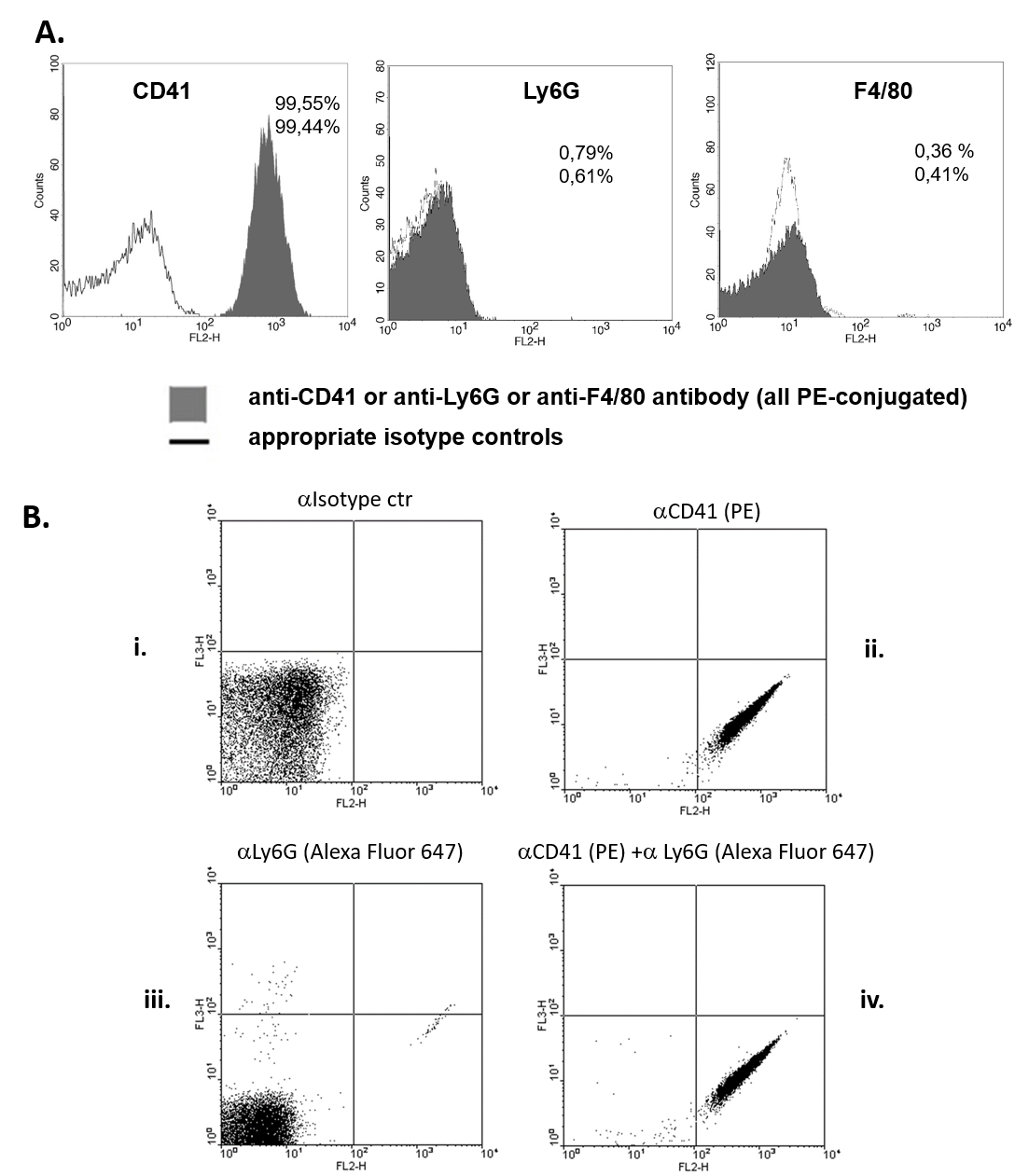


**Supplementary Figure S9.** Representative data on verification of platelet purity in transfer experiments. (**A**) Representative flow cytometry histograms of signal detection for CD41 (platelets), Ly6G (neutrophils), and F4/80 (monocytes/macrophages). Positive cell quantification is given from two repetitions. The number of CD41^+^ cells was always >99%. (**B**) Exemplary dot plots of double staining for CD41 and Ly6G in the same sample: (Bi) signal from a representative isotype control, (Bii) single staining for CD41, (Biii) single staining for Ly6G, (Biv) double staining for CD41 and Ly6G. Either PE (FL-2) or Alexa 647 (FL-3) conjugated antibodies were used.

**Supplementary Video S1.** Neutrophil extracellular trap (NET) formation in liver sinusoids of lean (ND) and obese (HFD) mice during endotoxemia (24 h post i.p. LPS). Neutrophils were labeled with PE-anti-Ly-6G antibody (red) and neutrophil elastase with Alexa Fluor 647 anti-NE (violet). Autofluorescent hepatocytes are green. The scale bar indicates 50 μm.

**Supplementary Video S2.** Neutrophil extracellular trap (NET) formation in liver sinusoids of lean (wt) and obese (ob/ob) mice during endotoxemia (24 h post i.p. LPS). Neutrophils were labeled with Brilliant Violet 421-anti-Ly-6G antibody (false red color) and neutrophil elastase with Alexa Fluor 647 anti-NE (violet). Autofluorescent hepatocytes are green. The scale bar indicates 50 μm.

**Supplementary Video S3.** Neutrophils in the vasculature of the adipose tissue of lean (ND) and obese (HFD) mice. Neutrophils were labeled with Brilliant Violet 421-anti-Ly-6G antibody (blue) and platelets with PE anti-CD49b (red). Autofluorescent adipose tissue is visible in white-green. The scale bar indicates 50 μm.

**Supplementary Video S4.** Interactions of platelets with neutrophils in liver sinusoids as captured with IVM. Neutrophils were labeled with Brilliant Violet 421-anti-Ly-6G antibody (blue) and platelets with PE anti-CD49b (red). The scale bar indicates 50 μm.
